# CCL5/CCR5/CYP1A1 pathway prompts liver cancer cells to survive in the combination of targeted and immunological therapies

**DOI:** 10.1111/cas.16320

**Published:** 2024-08-25

**Authors:** Yafei Wang, Biao Gao, Tianyu Jiao, Wenwen Zhang, Huizhong Shi, Hao Jiang, Xuerui Li, Junfeng Li, Xinlan Ge, Ke Pan, Chonghui Li, Guankun Mao, Shichun Lu

**Affiliations:** ^1^ Nankai University School of Medicine, Nankai University Tianjin China; ^2^ Faculty of Hepato‐Pancreato‐Biliary Surgery Chinese PLA General Hospital Beijing China; ^3^ Institute of Hepatobiliary Surgery of Chinese PLA Beijing China; ^4^ Key Laboratory of Digital Hepatobiliary Surgery of Chinese PLA Beijing China

**Keywords:** bergamottin, CCL5/CCR5/CYP1A1, combination therapy, HCC, lenvatinib

## Abstract

Combination therapy of anti‐programmed cell death protein‐1 (PD‐1) antibodies and tyrosine kinase inhibitors (TKIs) has significantly improved the prognosis for hepatocellular carcinoma (HCC), but many patients still have unsatisfactory outcomes. CD8 T cells are known to exert a pivotal function in the immune response against tumors. Nevertheless, most CD8 T cells in HCC tissues are in a state of exhaustion, losing the cytotoxic activity against malignant cells. Cytokines, mainly secreted by immune cells, play an important role in the occurrence and development of tumors. Here, we demonstrated the changes in exhausted CD8T cells during combination therapy by single‐cell RNA sequencing (scRNA‐seq) analysis on tumor samples before and after treatment. Combination therapy exerted a substantial impact on the exhausted CD8T cells, particularly in terms of cytokine expression. CCL5 was the most abundantly expressed cytokine in CD8T cells and exhausted CD8T cells, and its expression increased further after treatment. Subsequently, we discovered the CCL5/CCR5/CYP1A1 pathway through RNA sequencing (RNA‐seq) on CCL5‐stimulated Huh7 cells and verified through a series of experiments that this pathway can mediate the resistance of liver cancer cells to lenvatinib. Tissue experiments showed that after combination therapy, the CCL5/CCR5/CYP1A1 pathway was activated, which can benefit the residual tumor cells to survive treatment. Tumor‐bearing mouse experiments demonstrated that bergamottin (BGM), a competitive inhibitor of CYP1A1, can enhance the efficacy of both lenvatinib and combination therapy. Our research revealed one mechanism by which hepatoma cells can survive the combination therapy, providing a theoretical basis for the refined treatment of HCC.

## Graphical abstract

Hepatocellular carcinoma (HCC) tissue possessed a stronger metabolic reprogramming capacity for lenvatinib compared with liver tissue during the combination theapy based on anti‐PD1 antibody plus lenvatinib, as reflected by the activation of the CCL5/CCR5/CYP1A1 pathway. CYP1A1 inhibitor could improve the efficacy of lenvatinib or combination therapy for HCC.

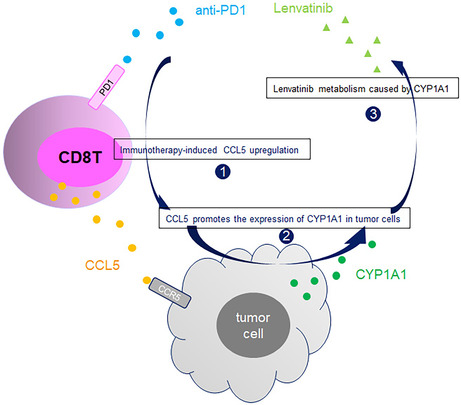

**Abbreviations**
BGM: bergamottin
CYP450: cytochrome P450
HCC: hepatocellular carcinoma
M2‐TAMs: M2‐type tumor‐associated macrophages
PD‐1: anti‐programmed cell death protein‐1
RNA‐seq: RNA sequencing
scRNA‐seq: single‐cell RNA sequencing
TKIs: tyrosine kinase inhibitors

## 1 INTRODUCTION

Hepatocellular carcinoma (HCC) ranks as the sixth most prevalent malignant tumor and stands as the fourth leading cause of cancer‐related deaths worldwide. Due to its insidious onset and rapid progression, most patients lose the opportunity for radical surgery, resulting in a poor prognosis.^1^, ^2^, ^3^ CD8 T cells are known to exert a pivotal function in the immune response against tumors. Nevertheless, most CD8 T cells in HCC tissues are in a state of exhaustion or suppression, losing the cytotoxic activity against malignant cells.^4^, ^5^, ^6^ Immunotherapy represented by immune checkpoint inhibitors (ICIs), exerts antitumor properties by revitalizing exhausted CD8 T cells and augmenting their cytotoxicity.^7^, ^8^ The advent of ICIs has significantly changed the treatment pattern for HCC; however, the effectiveness of a single drug is limited.^9^ Therefore, combination therapies based on ICIs have been widely studied.^10^, ^11^, ^12^, ^13^, ^14^, ^15^, ^16^ Studies indicate that tyrosine kinase inhibitors (TKIs) have the potential to enhance the antitumor efficacy of ICIs through diverse mechanisms such as normalizing tumor capillaries, enhancing immune cell infiltration and activation, reversing immunosuppression state within the tumor microenvironment (TME), and facilitating the release and presentation of tumor antigens.^15^, ^17^, ^18^, ^19^ One of our clinical trials showed that combination of PD‐1 inhibitor with lenvatinib as conversion therapy could benefit unresectable advanced HCC patients to achieve curative surgery, with a specific conversion rate of 30.6%.^20^ Although the combination of ICIs and TKIs have greatly improved the prognosis of patients with HCC, approximately half of them still encounter unsatisfactory outcomes.^12^, ^14^, ^21^ Hence, it is of utmost significance to elucidate the mechanism by which liver cancer cells survive the combination therapy.

Immune cells can synthesize and release a wide range of cytokines, exhibiting a multitude of significant biological functions.^22^ Cytokines are classified into six distinct categories based on their different biological functions: interleukin (IL), interferon (IFN), tumor necrosis factor (TNF), colony‐stimulating factor (CSF), growth factor(GF), and chemokine.^22^ Studies have shown that cytokines promote cellular interactions within the TME and are involved in many tumor‐related processes such as tumor formation, angiogenesis, immune responses, invasion, metastasis, and other pathological events, thus exhibiting multifaceted and complex functions in either promoting or inhibiting tumor progression.^23^, ^24^, ^25^, ^26^, ^27^, ^28^, ^29^ Recent studies have shown that cytokines are responsible for the resistance of tumor cells to anticancer medicines. IL‐6, 8, and 11 have been observed to facilitate the survival and proliferation of gastric cancer cells and play a role in mediating their resistance to chemotherapy, including oxaliplatin.^30^, ^31^ CC chemokine ligand‐2 (CCL2) has been found to not only elicit the insensitivity of tumor cells to chemotherapy and hormone therapy but also induce the resistance of tumor cells toward immunotherapy and targeted drugs such as sorafenib.^32^ M2‐type tumor‐associated macrophages (M2‐TAMs)‐derived tumor growth factor beta 1 (TGF‐β1) can diminish the responsiveness of esophageal squamous cell carcinoma (ESCC) cells to cisplatin, and knockdown of TGF‐β1 could reverse this effect.^33^ Therefore, we are interested in the alterations in cytokine expression exhibited by CD8 T cells in the context of combination therapy, as well as the consequential impact of such changes on the residual tumor cells.

To elucidate the underlying mechanism, we conducted single‐cell RNA sequencing (scRNA‐seq) on five HCC samples before or after combination therapy (anti‐PD1 antibody combined with lenvatinib). Our analysis revealed that CCL5 was the most highly expressed cytokine in both CD8 T cells and exhausted CD8 T cells, and there was a substantial rise during combination therapy. External datasets pertaining to melanoma or HCC suggested that immunotherapy is responsible for the upregulation of CCL5 expression. We then performed bulk RNA sequencing (RNAseq) on CCL5‐stimulated Huh7 cells and discovered the CCL5/CCR5/CYP1A1 pathway, which may prompt the drug resistance of hepatoma cells to lenvatinib. This pathway was subsequently validated by a series of cell‐ and tissue‐related experiments. Ultimately, animal experiments were undertaken to clarify the alterations in CYP1A1 expression within the liver or tumor tissues following the administration of anti‐PD1 antibody or/and lenvatinib. We also demonstrated that one CYP1A1 inhibitor, BGM, could improve the therapeutic efficacy of lenvatinib or combination therapy.

## 2 MATERIALS AND METHODS

### 2.1 Materials

#### 2.1.1 Reagents and materials

The reagents and materials used in our study are detailed in Table S1.

#### 2.1.2 Patients and specimens

The collection of pathological samples took place at the Chinese PLA General Hospital between October 2018 and September 2022. All patients provided their signatures on the written informed consent document. The medical information of patients was acquired from the electronic medical record system. In order to study the changes that occurred in tumor cells faced with combination therapy (anti‐PD1 antibody plus lenvatinb), tumor samples were selectively collected from the remaining viable parts of the surgical specimens from patients who received combination therapy.

### 2.2 Methods

#### 2.2.1 10X Genomics single‐cell data

Seven samples were obtained from four patients who underwent surgery following combination therapy (lenvatinib + camrelizumab, administered for five to seven cycles), including three puncture samples before combination therapy and four surgical specimens after combination therapy, to perfrom scRNA‐seq. The detailed methodology is shown in Appendix S2.

#### 2.2.2 Validation dataset

In order to validate our findings, we obtained gene sequencing data and corresponding clinical information from the GEO database. The validation sets used in our study included GSE211850, GSE153203, GSE149614, and GSE91061. The Cancer Genome Atlas (TCGA) database was additionally employed to validate our perspective.

#### 2.2.3 Cell culture, stimulation, and cell transfection

HepG2, Hep3B, Huh7, and Hepa 1–6 cells were cultured to validate the effect of CCL5 (100 ng/mL), maraviroc (MVC, 200 ng/mL), and lenvatinib (100 ng/mL) on CYP1A1 expression. SiCYP1A1 or CYP1A1 overexpression (CYP1A1 OE) was used to monitor its effect on the resistance of HCC cell lines to lenvatinib. The detailed methodology is shown in Appendix S2.

#### 2.2.4 RNA‐sequencing (RNA‐seq) analysis

CCL5‐stimulated Huh7 cells were used to perfrom RNA‐seq analysis. The detailed methodology is shown in Appendix S2.

#### 2.2.5 Quantitative real‐time PCR (qPCR)

After different stimulations for 24 h, the RNA of the cell lines was extracted using the RNAsimple Total RNA Kit. A total of 24 frozen tumor samples, including 12 samples after combination therapy and 12 specimens of direct surgeries, were swiftly homogenized upon the addition of lysis buffer, facilitating the extraction of RNA. The detailed methodology for qCPR is shown in Appendix S2.

The forward and reverse primers used in this study are detailed in Table S2.

#### 2.2.6 Western blotting (WB) analysis

Forty‐eight hours after different stimulations, the cell lines were washed with PBS and lysed using RIPA lysis buffer for protein extraction. A total of 25 frozen tumor samples, including 13 samples after combination therapy and 12 specimens of direct surgeries, were homogenized and lysed with RIPA lysis buffer. The detailed methodology for WB is shown in Appendix S2.

Finally, Image J software was used to perform relative quantification analysis based on grayscale values obtained from protein bands.

#### 2.2.7 Immunohistochemistry (IHC)

A total of 12 pathologically confirmed moderately differentiated tumor specimens, including six after combination therapy and six of direct operations, were selected for IHC. The detailed methodology for IHC is shown in Appendix S2. Five randomly selected fields of view were observed and photographed under a light microscope.

Image J software was used to assess the positivity rate by calculating the percentage of positively stained areas.

#### 2.2.8 Multiplex immunohistochemistry/immunofluorescence (mIHC/IF)

A total of 24 formalin‐fixed paraffin‐embedded (FFPE) tissues, including eight tumor samples from direct surgery and 16 tumor specimens with different proportions of tumor remaining after combination therapy, were chosen for multiplex mIHC/IF. Following the provided instructions, these tissue were subjected to immunostaining using anti‐human antibodies targeting CD8, CCL5, PDCD1, and CYP1A1 with the Opal 6‐Plex Manual Detection Kit along with DAPI counterstaining. Images were acquired using the Vectra 3.0 Pathology Imaging System Microscope (Perkin‐Elmer) and analyzed using QuPath (v0.4.3).

#### 2.2.9 CCK‐8 kit assay, clone formation assay, and wound‐healing assay

These cell phenotype experiments were used to assess the proliferation, migration, and clone formation abilities of cell lines in different groups. The detailed methodology is shown in Appendix S2.

#### 2.2.10 Dual IF

Patient‐derived frozen tissue was used to perform dual IF. The detailed methodology is shown in Appendix S2.

#### 2.2.11 Mice model

A total of 2 × 10^6^ Hepa1–6 cells in 100 μL PBS were subcutaneously injected into 8‐week‐old male C57BL/6J mice weighing about 20 g. Tumor volume (length × width^2^ × ½) was assessed every 3 days. Once tumors reached 200 mm^3^, mice were randomly divided into groups to receive lenvatinib, bergamottin (BGM), anti‐PD1 antibody, anti‐PD1 + BGM, lenvatinib + BGM, lenvatinib + anti‐PD1 antibody, lenvatinib + anti‐PD1 antibody + BGM, or corresponding solvents. Lenvatinib was dissolved in 3% hydrochloric acid, while BGM was firstly dissolved in 10% DMSO and then diluted with 90% corn oil. Anti‐PD1 and IgG antibodies were diluted with PBS. The drugs were administered as follows: oral gavage of 5 mg/kg lenvatinib daily; intraperitoneal injection of 5 mg/kg anti‐PD1 antibody or IgG every 3 days; and intraperitoneal injection of 50 mg/kg BGM daily. The experiment was planned to be terminated on day 15 after intervention for specimen collection. All animal studies adhered to the guidelines established by the institution's ethical committee.

#### 2.2.12 Statistical analysis

Numerical variables were presented as mean ± SD, and differences between groups were assessed using appropriate statistical tests such as Student's *t*‐test, one‐way analysis of variance, or Mann–Whitney *U* test. Categorical variables were reported as *n* (%), and the comparison between groups was conducted using the Pearson *χ* ^2^ test and Fisher's exact test. The sequencing data were analyzed using R software. qPCR, WB, IHC, mIHC, and clinical data were analyzed largely based on Graphpad Prism V.8.0. A two‐tailed *p*‐value <0.05 was considered statistically significant.

## 3 RESULTS

### 3.1 scRNA‐seq revealed the cytokine expression changes of exhausted CD8 T cells during the combination therapy

**FIGURE 1:**
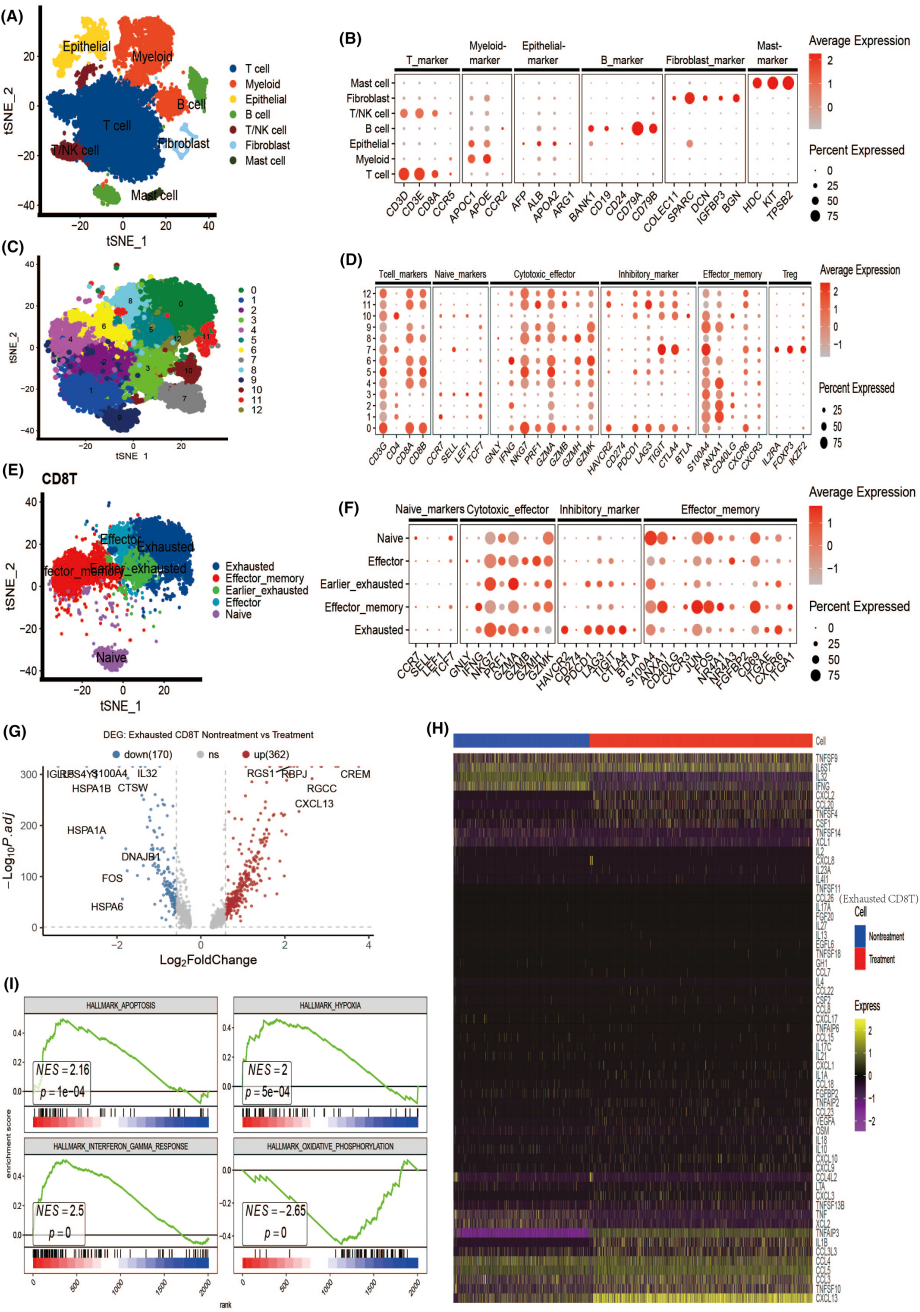
There were significant changes in exhausted CD8T cells before and after combination therapy, especially in the expression of cytokines. (A) t‐SNE plot showing the clustering results of seven major cell types for 34,512 high‐quality single cells from samples before or after combination therapy. (B) Dot plot showing the highly expressed marker genes in each major cell type. (C) t‐SNE plot showing the clustering results of T cell. (D) Dot plot showing the highly expressed marker genes in each T cell cluster. (E) t‐SNE plot showing the clustering results of five major CD8T cell types. (F) Dot plot showing the highly expressed marker genes in each CD8T cell type. (G) Volcano plot showing the differentially expressed genes (DEGs) of exhausted CD8T cells before and after combination therapy. (H) Heatmap showing the different expressions of cytokine‐related genes in exhausted CD8T cells before and after combination therapy. (I) Gene set enrichment analysis shows enrichment pathways of exhausted CD8T cells before and after combination therapy.

After filtering the scRNA‐seq data from the seven samples, a total of 34,512 high‐quality cells were obtained, comprising 18,969 pre‐combination therapy cells and 15,543 post‐combination therapy cells. Following data preprocessing, sample integration, and principal component analysis (PCA), eleven distinct cell clusters were identified (Figure S1). Based on the expression patterns of well‐known marker genes, these 11 clusters were classified into immune cell types (T cells, B cells, T/NK cells, myeloid cells) and non‐immune cell types (mast cells, epithelial cells, and fibroblasts) (Figure 1A). The expression profiles of marker genes in each cell type are depicted in Figure 1B. We further extracted the T cells to perform secondary dimensionality reduction and discovered that T cells could be subdivided into 13 cell clusters (Figure 1C). Cell clusters 0, 4, 5, 6, 8, 9, 11, and 12 exhibited overexpression of CD8 T cell markers (CD3G, CD8A, and CD8B) while underexpressing CD4; thus, they were classified as CD8T cells (Figure 1D). Among them, cell clusters 0, 11, and 12 showed elevated expression of immunosuppression‐related genes including hepatitis A virus cellular receptor 2 (HAVCR2), programmed cell death protein 1 (PDCD1), lymphocyte activation gene 3 (LAG3), inhibitory receptor T cell immunoreceptor with Ig and ITIM domains (TIGIT), and cytotoxic T lymphocyte‐associated antigen 4 (CTLA4), indicating their status as exhausted CD8T cells (Figure 1E). Cell clusters 4 and 6 overexpressed marker genes for effector‐memory CD8 T cells, including S100A4, ANXA1, JUN, FOS, and CD69; hence, they were annotated as effector memory CD8 T cells (Figure 1E). Other subtypes of CD8 T cells were also classified based on the expression patterns of corresponding marker genes (Figure 1E,F).

Differentially expressed genes in response to combination therapy were identified using the Limma package in the R software. The volcano plot showed that after combination therapy, a total of 362 genes were upregulated and 170 genes were downregulated (Figure 1G). We then extracted cytokine‐related genes from the GeneCards website (https://www.genecards.org/Search/Keyword?queryString=cytokine) to compare the differences in cytokine expression by exhausted CD8T cells before and after combination therapy. Combination therapy dramatically upregulated the expression of many cytokine genes in exhausted CD8 T cells, as shown by the heatmap analysis (Figure 1H). Comparison of CD8 T cells before and after therapy using gene set enrichment analysis (GSEA) indicated significant differences in the APOPTOSIS, HYPOXIA, INTERFERON_GAMMA, and OXIDATIVE_PHOSPHORYLATION pathways (Figure 1I). These results demonstrated that combination therapy exerted a substantial impact on the exhausted CD8T cells, particularly in terms of cytokine expression.

In addition, we further analyzed the differences in the expression of cytotoxic, immunosuppressive genes of exhausted CD8T cells before and after combination therapy. We found that cytotoxic genes of exhausted CD8T cells, such as recombinant perforin 1 (PRF1) and granzyme B (GZMB), tended to increase, whereas granzyme A (GZMA) and gamma‐interferon (IFNG) tended to decrease after combination therapy (Figure S2A–D). Immunosuppressive genes of exhausted CD8 T cells, such as T cell immunoglobulin structural domain and mucin domain‐3 (TIM‐3) and CTLA4, tended to increase after combination therapy, whereas PDCD1 and LAG3 showed no significant changes (Figure S2E–H). These results reflect the complex changes that occur in exhausted CD8T cells during combination therapy. They also suggest that individual marker genes (e.g., PDCD1) are insufficient for assessing CD8T cell function.

### 3.2 Anti‐PD1 therapy induced exhausted CD8 T cells to express more CCL5 during combination therapy

**FIGURE 2:**
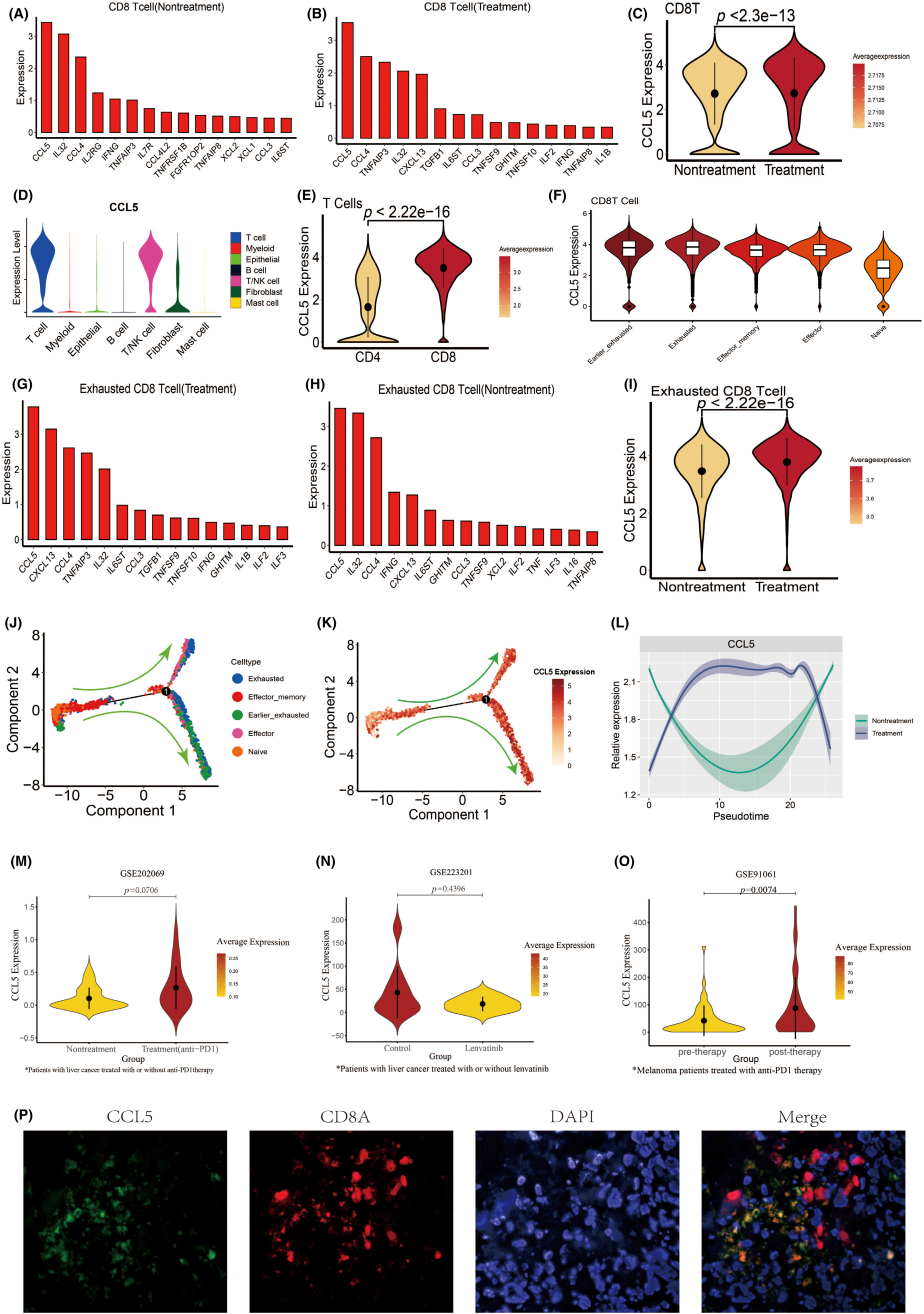
The increased CCL5 after combination therapy was mainly derived from exhausted CD8T cells. (A) Top 15 cytokine‐related genes overexpressed by CD8T cells before combination therapy. (B) Top 15 cytokine‐related genes overexpressed by CD8T cells after combination therapy. (C) Violin plots showing the expression level of CCL5 in CD8T cells before and after combination therapy. (D) Violin plots showing the expression level of CCL5 in seven major cell types in the tumor microenvironment (TME) of hepatocellular carcinoma (HCC). (E) Violin plots showing the expression level of CCL5 in CD4 T cells and CD8 T cells. (F) Violin plots showing the expression level of CCL5 in five major CD8T cell types. (G) Top 15 cytokine‐related genes overexpressed by exhausted CD8T cells after therapy. (H) Top 15 cytokine‐related genes overexpressed by exhausted CD8T cells before therapy. (I) Violin plots showing the expression level of CCL5 in exhausted CD8T cells before and after combination therapy. (J) The developmental trajectory of CD8 T cells inferred by Monocle2. (K) 2D pseudotime plot showing the dynamics of CCL5 expression in CD8T cells. (L) Shaded line plot indicating the expression levels of CCL5 along the pseudotime in CD8T cells before and after combination therapy. (M) Violin plots showing the expression level of CCL5 in the anti‐PD1 therapy group and the nontreatment group in the GSE202069 dataset. (N) Violin plots showing the expression level of CCL5 in the lenvatinib group and the control group in the GSE223201 dataset. (O) Violin plots showing the expression level of CCL5 in the post‐therapy group and the pre‐therapy group in the GSE91061 dataset. (P) Dual immunofluorescence showing colocalization of CCL5 and CD8A in the TME.

Regardless of whether the combination therapy was conducted or not, CCL5 was the most highly expressed cytokine of CD8T cells (Figure 2A,B). Combination therapy resulted in a further upregulation in CCL5 expression among CD8T cells (Figure 2C). To determine the source of CCL5, we investigated CCL5 expression levels across cell types in the TME and found that T cells had the highest CCL5 expression level (Figure 2D). CCL5 expression was significantly increased in CD8 T cells compared with CD4 T cells (Figure 2E), and exhausted CD8 T cells expressed the most CCL5 (Figure 2F). Similar to CD8 T cells, the most expressed cytokine in exhausted CD8 T cells was also CCL5, and their CCL5 expression was significantly upregulated after combination therapy (Figure 2G–I).

The relationship between CCL5 expression and CD8 T cell differentiation trajectories was then investigated. Naive_CD8T and Effector_memory CD8T cells were at the beginning of differentiation, Effector_CD8T cells were in the middle of differentiation, and early‐exhausted CD8+ T cells and exhausted CD8+ T cells were at the end of differentiation, indicating that CD8T cells in the TME undergo an initially immature state, an intermediate effect state, and then gradually develop into an exhausted phenotype (Figure 2J). We demonstrated CCL 5 expression on the trajectory map and discovered that CCL 5 expression increased as CD8T cells were exhausted (Figure 2K). After combination therapy, CD8 T cells expressed more CCL5 at most stages of differentiation than before therapy (Figure 2L).

We obtained and processed RNA‐seq data from the GEO database to demonstrate whether the upregulation of CCL5 expression was predominantly related to anti‐PD1 treatment or lenvatinib. The violin plot showed that only the anti‐PD1 therapy could increase the CCL5 expression in HCC tissue, while lenvatinib could not (Figure 2M,N). CCL5 expression was also found to be upregulated by anti‐PD1 therapy in melanoma (Figure 2O). These analyses suggested that the anti‐PD1 antibody was the driving force behind the elevation of CCL5 expression during combination therapy. Consistent with the results of the bioinformatics analysis outlined before, dual IF staining confirmed colocalization of CD8A and CCL5 signals in HCC tissue. (Figure 2P).

### 3.3 CCL5 promoted the expression of lenvatinib resistance genes in liver cancer cells

**FIGURE 3:**
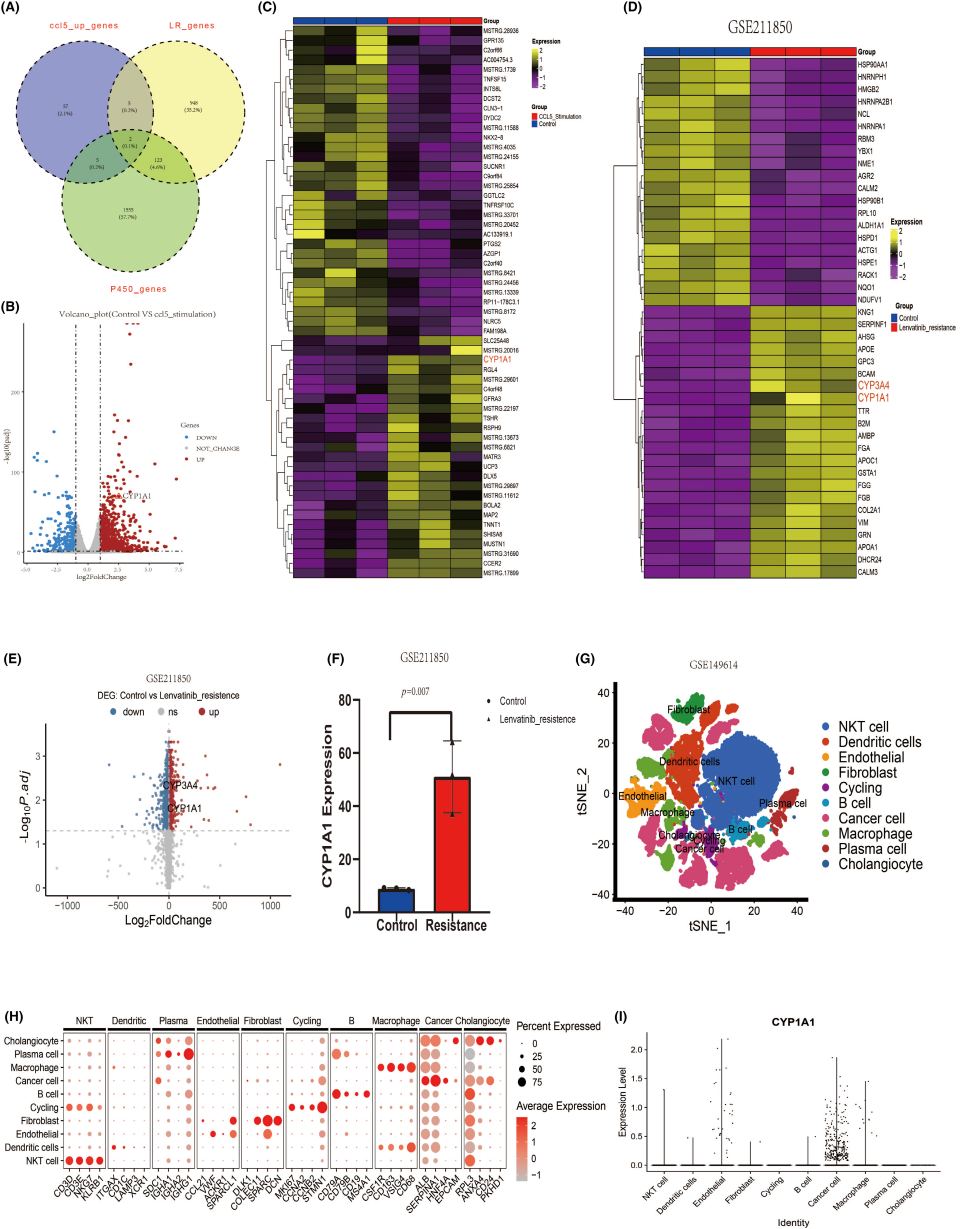
CCL5 induced tumor cells to overexpress the lenvatinib resistance gene CYP1A1. (A) Venn diagram showing intersecting genes in the upregulated genes of the CCL5‐stimulated Huh7 cells, lenvatinib resistance genes, and cytochrome P450 enzymes‐related genes. (B) Volcano plot showing DEGs between the control and CCL5 stimulus group of Huh7 cells. (C) Heatmap showing up‐ and downregulated genes in the CCL5 stimulus group of Huh7 cells. (D) Heatmap showing up‐ and downregulated genes in the lenvatinib‐resistant Huh7 cells in the GSE211850 dataset. (E) Volcano plot showing DEGs between the control and lenvatinib‐resistant groups in the GSE211850 dataset. (F) Differences in CYP1A1 expression in the control and lenvatinib‐resistant groups in the GSE211850 dataset. (G) t‐SNE plot showing the clustering results of 10 major cell types in the GSE149614 dataset. (H) Dot plot showing the highly expressed marker genes in each major cell type in the GSE149614 dataset. (I) Violin plots showing the expression level of CYP1A1 in 10 major cell types in the GSE149614 dataset.

Given the significant changes in CCL5 expression during combination therapy, we were intrigued about its effect on residual viable tumor cells. Therefore, we conducted RNA‐seq on CCL5‐stimulated Huh7 cells. We performed differential analysis of gene expression between the CCL5‐stimulated group and the control group using the “DESeq” package of R software. Using the criteria |logFC >0.58| and *p*.adjust <0.05, we screened for the differentially expressed genes. Among the upregulated genes, we found some cytochrome P450 enzymes (CYPs). So we were curious whether these CYPs had an impact on the efficacy of lenvatinib. We downloaded bulk‐RNAseq data of the lenvatinib‐resistant Huh7 cell line from the GEO database. Genes related to the CYPs were also downloaded from GeneCards for further study. By selecting the intersection of these three sets of genes, we were able to identify two consistently upregulated intersection genes, and we were interested in one of them, CYP1A1 (Figure 3A). Volcano plot and heatmap revealed that the expression of CYP1A1 in Huh7 cells stimulated by CCL5 is significantly higher than that in the control group (Figure 3B,C). Figure 3D,E depicted the differential expression of two important lenvatinib metabolism‐related genes, CYP1A1 and CYP3A4, in lenvatinib‐resistant Huh7 cells versus normal cells. The expression of CYP1A1 was substantially upregulated in lenvatinib‐resistant Huh7 cells, as shown in Figure 3F.

The scRNA‐seq data of HCC was obtained from the GEO database to investigate the expression pattern of CYP1A1 across various cell types inside the TME. Following the dimensionality reduction and clustering of the filtered cells, we proceeded to annotate these cells by utilizing marker genes, as depicted in Figure 3G,H. As illustrated, the expression of CYP1A1 was primarily identified in cancer cells, while limited expression was detected in other cell types (Figure 3I).

Based on these findings, we hypothesized that CCL5 may play a role in levatinib resistance by increasing CYP1A1 expression in tumor cells. Since CCR5 is the main receptor of CCL5,^34^ we next conducted a series of experiments to investigate the regulatory effect of the CCL5/CCR5 axis on CYP1A1 expression.

### 3.4 The CCL5/CCR5/CYP1A1 pathway regulated the resistance of liver cancer cells to lenvatinib

**FIGURE 4:**
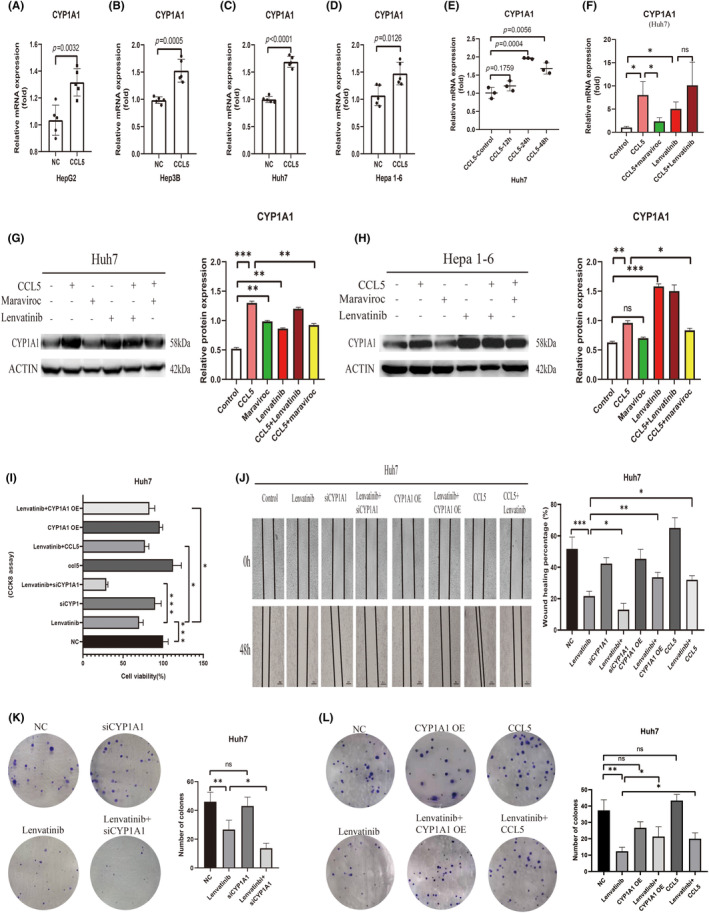
The resistance of liver cancer cells to lenvatinib is regulated by the CCL5/CCR5/CYP1A1 pathway. (A–D) The mRNA level of CYP1A1 in different cell lines (human hepatocellular carcinoma [HCC] cell lines Huh7, HepG2, and Hep3B, as well as the murine HCC cell line Hepa1‐6) before and after CCL5 stimulation. (E) The mRNA level of CYP1A1 in the Huh7 cell line stimulated by 100 ng/mL CCL5 after 0, 12, 24, and 48 h. (F) The mRNA level of CYP1A1 in Huh7 cells in groups with different stimulations after 24 h: control group, CCL5 group, CCL5 + maraviroc group, lenvatinib group, and CCL5 + lenvatinib group. (G, H) The protein level of CYP1A1 in Huh7 cells in groups with different stimulation after 48 h: control group, CCL5 group, maraviroc group, lenvatinib group, CCL5 + lenvatinib group, and CCL5 + maraviroc group. CCK‐8 assay (I), wound‐healing assay (J), Clone formation assay (K, L) showing that siCYP1A1 could improve the killing effect of lenvatinib on tumor cells. CYP1A1 OE: CYP1A1 over‐expression group. *p < 0.05, **p < 0.01, ***p < 0.001.

Human HCC cell lines Huh7, HepG2, and Hep 3B, as well as the murine HCC cell line Hepa 1–6, were chosen to examine the effect of CCL5 on CYP1A1 expression. After stimulation with CCL5, the mRNA expression of CYP1A1 in these cells exhibited an upward trend (Figure 4A–D). The Huh7 cell line was chosen for the experiment that followed. We found that the mRNA expression of CYP1A1 was substantially upregulated 24 h after stimulation with 100 ng/mL CCL5. However, 48 h after stimulation, CYP1A1 expression did not increase further (Figure 4E). In addition, we observed that CYP1A1 expression in Huh7 cells increased with the stimulating concentration of CCL5 (Figure S3). Maraviroc (MVC), a specific antagonist of CCR5, could reverse the promotion effect of CCL5 on CYP1A1 (Figure 4F). Similar to CCL5, lenvatinib stimulation also led to an increase in CYP1A1 expression in Huh7 cells (Figure 4F). WB experiments revealed that although both CCL5 and MVC alone could cause the upregulation of CYP1A1, MVC could instead downregulate the upregulation of CYP1A1 caused by CCL5 when both were used simultaneously (Figure 4G). Lenvatinib can also increase the expression of the CYP1A1 protein in Huh7 cells (Figure 4G). The Hepa 1–6 cell line yielded identical experimental outcomes (Figure 4H).

SiRNA was used to inhibit CYP1A1 expression in Huh7 cells, and the knockdown effect was confirmed by qPCR and WB analysis (Figure S4). The CCK‐8 assay revealed that si‐CYP1A1 can augment the lethal effect of lenvatinib on Huh7 cells, whereas CCL5 or CYP1A1 overexpression (CYP1A1 OE) had the opposite effect (Figure 4I). The wound‐healing assays and colony formation assays demonstrated similar results (Figure 4J–L).

These results confirmed that the CCL5/CCR5/CYP1A1 pathway has a regulatory influence on lenvatinib's capacity to inhibit tumor cells. Consequently, we conducted experiments at the tissue level to demonstrate the alterations of this pathway in residual tumor tissue following combination therapy.

### 3.5 After combination therapy, the expression of CCL5, CCR5, and CYP1A1 increased in the remaining tumor tissues

**FIGURE 5:**
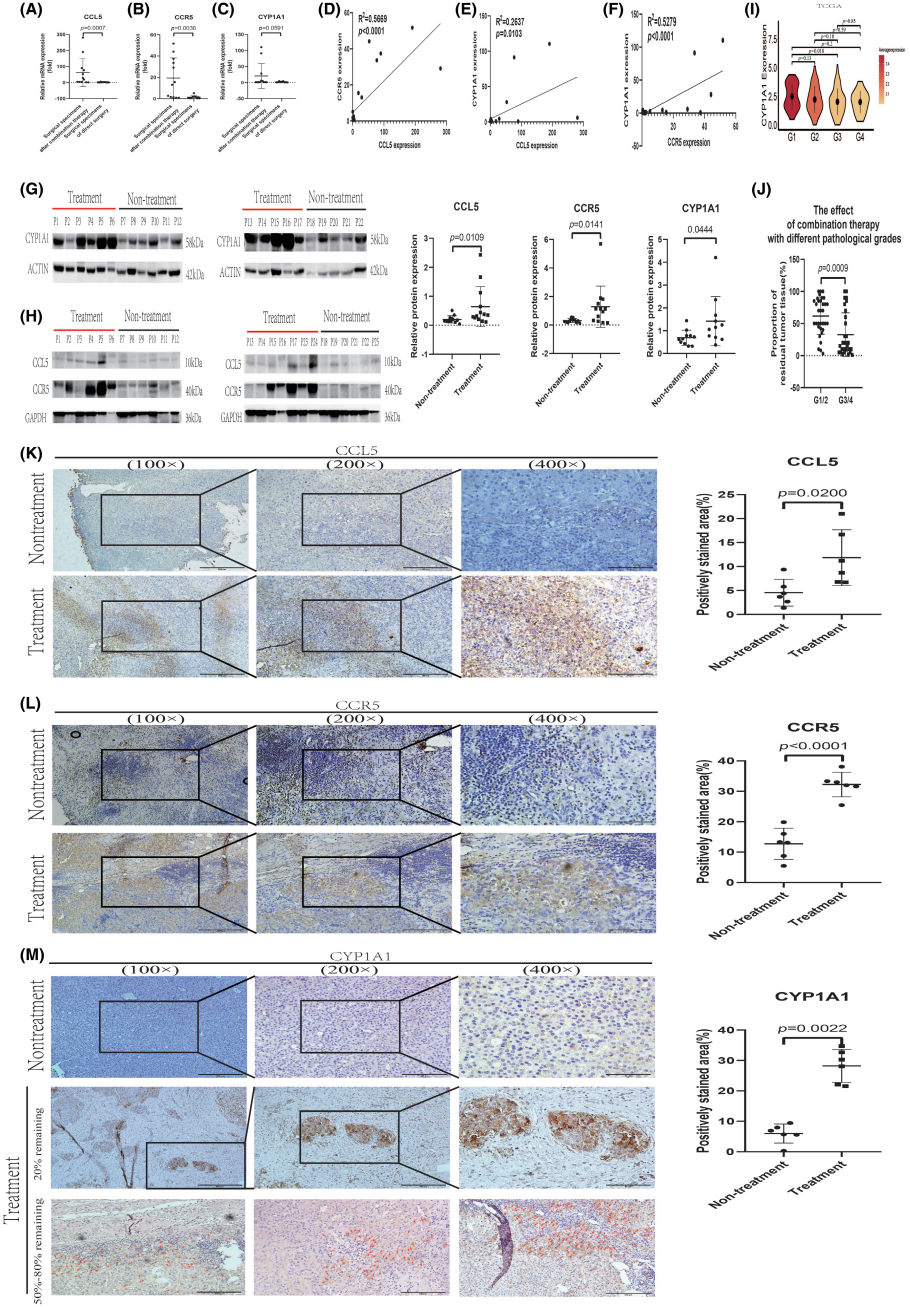
The CCL5/CCR5/CYP1A1 pathway was activated in the remaining tumor tissue after combination therapy. (A–C) mRNA levels of CCL5, CCR5, and CYP1A1 in residual tumor tissue before and after combination therapy. (D–F) Correlation analysis of CCL5, CCR5, and CYP1A1 in residual tumor tissue after combination therapy. (G, H) Protein levels of CCL5, CCR5, and CYP1A1 in residual tumor tissue before and after combination therapy. (I) Hepatocellular carcinoma (HCC) bulk RNA‐seq data from the TCGA database showed the expression of CYP1A1 in specimens of different pathological grades. (J) The proportion of residual tumor cells in specimens with different degrees of pathological differentiation after combination therapy. (K–M) Representative immunohistochemical staining images of CCL5, CCR5, and CYP1A1 before and after combination therapy. The red arrows show the CYP1A1‐positive stained tumor cells.

The mRNA expression of CCL5, CCR5, and CYP1A1 in tumor specimens from surgery after combination therapy was significantly higher than that in tumor tissues from direct surgery (Figure 5A–C). Nevertheless, the expression of CYP3A4, a pivotal enzyme responsible for the metabolism of lenvatinib, did not exhibit any significant variations before and after treatment (Figure S5). Correlation analysis of the expression of CCL5, CCR5, and CYP1A1 in tumor tissues after combination therapy revealed a certain degree of correlation among them (Figure 5D–F). In accordance with the qPCR findings, the WB analyses demonstrated a notable elevation in the protein levels of CCL5, CCR5, and CYP1A1 within the cancer tissues after combination therapy (Figure 5G,H).

We analyzed RNA‐seq data from 371 HCC patients in the TCGA database and found that as the pathological grades increased (i.e., the degrees of differentiation decreased), the expression of CYP1A1 in tumor tissue decreased (Figure 5I). This makes sense because CYP1A1 is an enzyme expressed by normal liver cells, and its expression reduces when tumors are poorly differentiated. However, it should also be noted that the expression of CYP1A1 in the tumor tissues of a considerable number of patients was not lower or even higher than that in normal liver tissues (Figure S6). This reflects the heterogeneity and metabolic reprogramming of tumor cells in the TME. Sixty surgical specimens (detailed information is exhibited in Table S3) with residual tumors containing pathological differentiation information after combination therapy were analyzed to explore the relationship between pathological differentiation and the efficacy of combination therapy. We found that the proportion of residual tumors in poorly differentiated specimens was considerably lower than that in those highly or moderately differentiated samples (Figure 5J). This phenomenon can be reasonably explained by considering that poorly differentiated tumors express lower CYP1A1 and therefore have a weak ability to metabolize lenvatinib.

To rule out the influence of pathological differentiation on CYP1A1, we selected 12 samples with moderate differentiation for IHC (six surgical samples after combination therapy and six samples of direct surgery). The IHC results showed upregulated expression of CCL5, CCR5, and CYP1A1 in tumor samples from patients following combination therapy (Figure 5K–M). At the same time, we observed the phenomenon of increased lymphocyte infiltration after treatment (Figure 5K–M). CCL5 was mainly expressed in the tertiary lymphoid structure (TLS), whereas CYP1A1 was predominantly expressed in the remaining active tumor cells, especially those adjacent to TLS (Figure 5K,M).

### 3.6 After combination therapy, more CD8 ^+^ CCL5 ^+^ T cells infiltrated into the TME, which led to high expression of CYP1A1 in residual tumor cells

**FIGURE 6:**
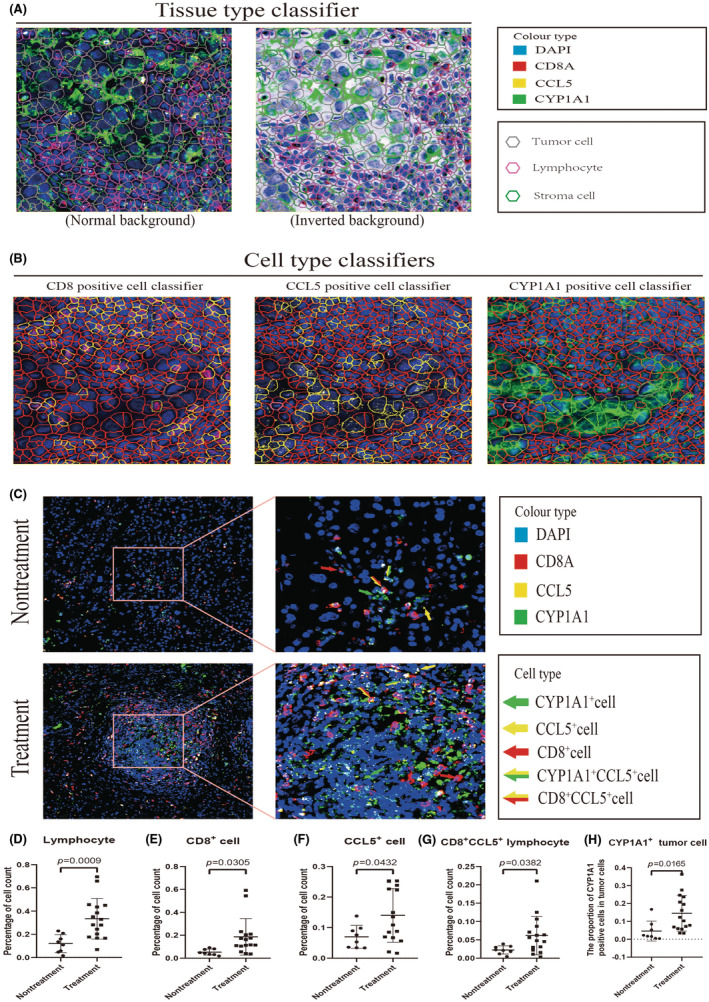
The multiplex immunohistochemistry (mIHC) demonstrated alterations in the infiltration of CD8T cells and the expression levels of CCL5 and CYP1A1 within residual tumor tissue following combination therapy. (A) Tissue type classifier trained by “QuPath” based on the following parameters: Nucleus area/perimeter/circularity/max caliper/min caliper/eccentricity/DAPI mean/DAPI sum/DAPI std dev/DAPI max/DAPI min/DAPI range, Cell area/perimeter/circularity/max caliper/min caliper/eccentricity, and nucleus/cell area ratio. (B) The positive cell classifier trained by “QuPath” based on the “cell opal mean” on different fluorescent channels. (C) Representative images of mIHC showing single‐positive or double‐positive cells before and after combination therapy. (D–H) Percentages of lymphocytes, CD8+ cells, CCL5+ cells, CD8+CCL5+ lymphocytes, and CYP1A1+ tumor cells before and after combination therapy.

To analyze the impacts of combination therapy on the infiltration of CD8T cells in the TME and the expression of CCL5 and CYP1A1 in residual tumor tissue, we stained the slices of 24 specimens with mIHC (eight samples obtained from direct surgery and 16 surgical samples obtained after combination therapy). “QuPath” software was used to analyze the mIHC images. Non‐necrotic, well‐stained regions were delineated manually. After cell detection, appropriate parameters were chosen to train tissue classifiers and positively stained cell classifiers (Figure 6A,B). From the images, we can see that CYP1A1 was predominantly expressed in tumor cells, while other cells barely expressed it (Figure 6A). Regardless of whether patients received combination therapy or not, the phenomenon of CYP1A1^+^ tumor cells gathering around CD8^+^CCL5^+^ lymphocytes was frequently observed (Figure 6C). Numerous cells costained for CD8A and CCL5 were observed, confirming the previously described notion that CD8T cells were the primary source of CCL5 (Figure 6B,C).

Tissue classifiers and cell classifiers were combined to create composite classifiers. After exporting the data measured by QuPath, we analyzed the data with R software and created graphs with GraphPad Prism 8.0 software. We discovered that the lymphocyte infiltration and the expression of CD8A and CCL5 increased in tumor tissue after combination therapy (Figure 6D–F). We also found that most CD8T cells expressed PD1 with or without combination therapy (Figure S7), a result consistent with scRNA‐seq analysis (Figure S2F). Meanwhile, the infiltration of CD8^+^CCL5^+^ lymphocytes increased (Figure 6G). After analyzing the CYP1A1 expression in residual tumor cells, we found that the proportion of CYP1A1 positively stained tumor cells increased considerably after treatment (Figure 6H).

These results suggested that during combination therapy, the remaining tumor cells can utilize CD8T cells‐derived CCL5 to express more CYP1A1 to resist lenvatinib.

### 3.7 Bergamottin, a competitive CYP1A1 inhibitor, can enhance the efficacy of lenvatinib or combination therapy in killing tumors

We had previously demonstrated that interfering with CYP1A1 can enhance the killing effect of lenvatinib on hepatoma cells. Here we conducted mouse experiments to investigate whether BGM, a competitive CYP1A1 inhibitor, can improve the efficacy of lenvatinib or combination therapy in vivo.

We conducted animal experiments in the following groups: control group, lenvatinib group, anti‐PD1 antibody group, BGM group, lenvatinib + BGM group, anti‐PD1antibody + BGM group, lenvatinib + anti‐PD1 antibody group, and lenvatinib + anti‐PD1 antibody + BGM group. The tumor volume was measured every 3 days. The experiment was terminated on day 15 after intervention for the collection of specimens.

**FIGURE 7:**
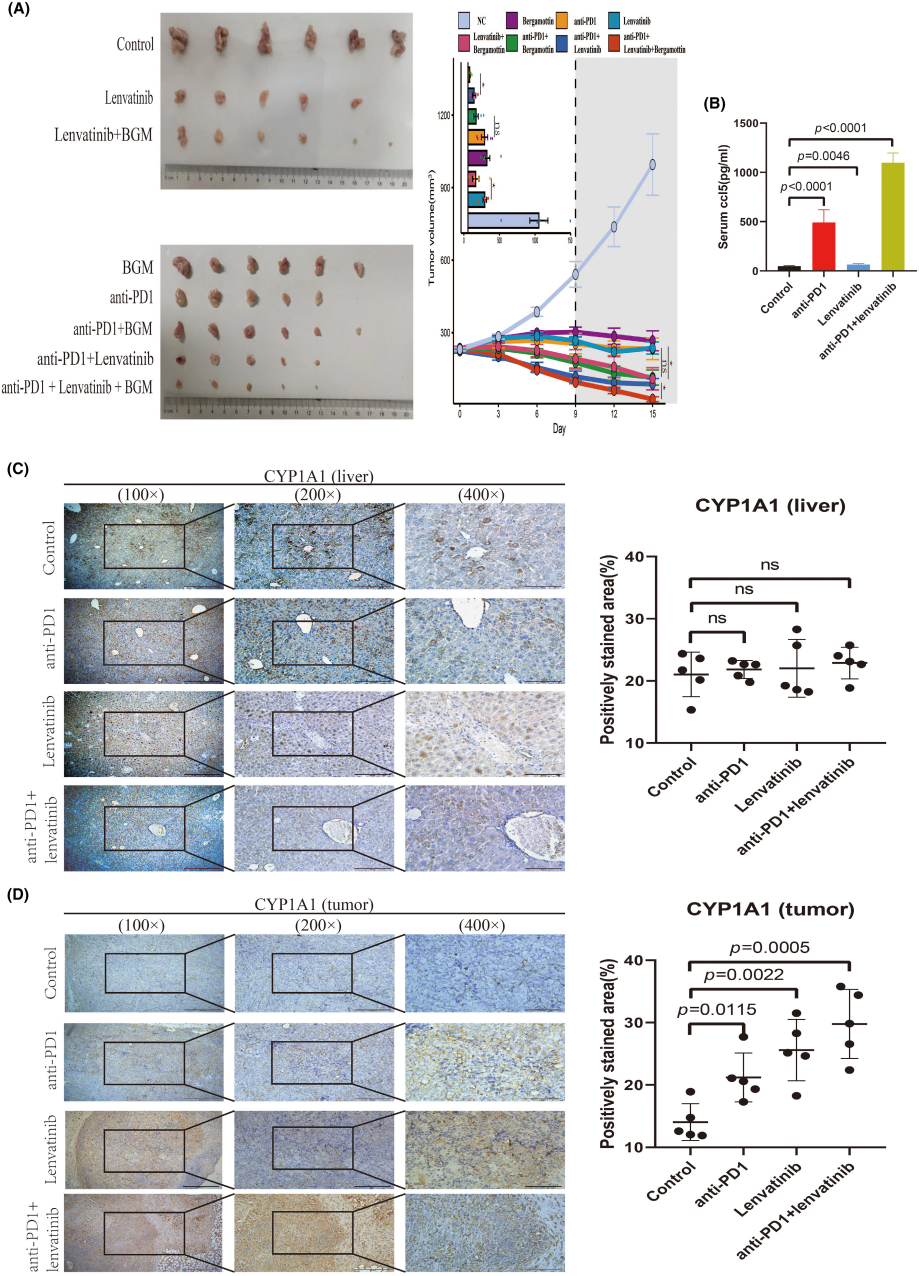
Experiments in tumor‐bearing mice demonstrated changes in peripheral blood CCL5 and liver and tumor CYP1A1 after anti‐PD1 or/and lenvatinib therapy. Inhibition of CYP1A1 improved the efficacy of lenvatinib or combination therapy. (A) Tumor volumes after 15 days of different interventions. (B) ELISA assays showing the concentrations of CCL5 in mouse serum after 15 days of different interventions. (C) Representative images of IHC showing CYP1A1 expression in the livers of mice treated with PD1 or/and lenvatinib. (D) Representative images of IHC showing CYP1A1 expression in the tumors of mice treated with PD1 or/and lenvatinib.

After subcutaneous tumor formation, six mice were assigned to each group, four mice died due to operational reasons during drug administration, and no mice died due to drug‐related adverse events. The line graph of tumor volume suggested that BGM can improve the efficacy of both lenvatinib and combination therapy (Figure 7A). ELISA revealed that the expression of CCL5 in the plasma of mice treated with anti‐PD1 was substantially elevated, whereas the effect of lenvatinib on CCL5 expression was insignificant (Figure 7B). When comparing the drug responsiveness of the liver and tumor, we found an intriguing phenomenon: The expression of CYP1A1 in tumor tissues exhibited significant intergroup differences, whereas no significant difference was observed in liver tissues (Figure 7C,D). While it was challenging to quantify the number of lymphocytes, it was clear that lenvatinib facilitated lymphocyte infiltration into the TME more than the anti‐PD1 antibody (Figure 7D), consistent with the findings of other researchers. In addition, we observed that the expression of CCL5, CD8A, and PD1 in the tumor tissue of mice in the combination therapy group was significantly higher than that in the control group (Figure S8), a result consistent with that of mIHC (Figure S7).

## 4 DISCUSSION

Uncovering the changes in tumor cells during combination therapy can provide insights for enhancing drug efficacy. Our study demonstrated the influence of one cytokine on the metabolism of hepatoma cells during combination therapy.

We conducted scRNA‐seq on residual tumor tissues before and after treatment and observed significant changes in CD8T cells, especially their cytokine expression. As the most highly expressed cytokine of CD8T cells, CCL5 had attracted our attention. CCL5, also known as RANTES, is a member of the chemokine subfamily CC. In addition to the T cell (the most important cell type that secretes CCL5), other cell types such as macrophage, eosinophil, fibroblast, epithelial cell, and so on can also secrete CCL5.^35^ Our study found that being the most important cells for tumor immunity, CD8T cells secreted more CCL5 compared with other cell types. As CD8 T cells gradually developed into an exhausted state, their CCL5 expression tended to increase, and the combination therapy led to a further increase in their CCL5 expression. Subsequent experiments validated the above findings. The upregulation of CCL5 expression in residual tumor tissue after treatment can be attributed to the increased release of CCL5 by CD8T cells stimulated by immunotherapy as well as the increased infiltration of CD8T cells into the TME caused by lenvatinib.^36^

C‐C chemokine receptor type 5 (CCR5), a G‐protein‐coupled receptor, is the most essential receptor for CCL5 because it has a higher affinity than CCR1, CCR3, and CCR4.^37^ The tumor‐promoting effect of the CCL5/CCR5 axis has been extensively studied in recent years. The CCL5/CCR5 axis can directly promote tumor formation, development, invasion, and metastasis, or it can indirectly aid in forming an immunosuppressive microenvironment or promote angiogenesis, tumor metabolism, chemotherapy resistance, and so on, thereby playing a crucial role in promoting tumor growth.^38^, ^39^, ^40^, ^41^, ^42^, ^43^, ^44^, ^45^ We performed RNAseq analysis on the CCL5‐stimulated Huh7 cell line, and in combination with the public RNAseq data on lenvatinib‐resistant Huh7 cell line we found that the CCL5/CCR5/CYP1A1 pathway may contribute to lenvatinib resistance in Huh7 cells. We then validated this pathway through in vitro experiments. IHC and mIHC further exhibited the increased infiltration of CD8^+^CCL5^+^ T cells after treatment, leading to an upregulated expression of CYP1A1 in residual tumor cells.

Cytochrome P450 (CYP450) enzymes, also known as P450s or CYPs, are a group of heme‐thiolate enzymes primarily recognized as mono‐oxygenases that catalyze a wide range of chemical transformations and act on diverse substrates.^46^ Previous studies have mainly focused on the effect of cytokines on liver CYP450 enzymes. Interleukin‐6 (IL‐6) may downregulate different CYP isoenzymes (notably CYP3A4) in chronic inflammatory patients.^47^ Through the upregulation of cytochrome P450 1B1 expression, TNF‐α amplifies the genotoxic effects of benzo[a]pyrene on rat liver epithelial cells.^48^ There is limited research on how cytokines affect the expression of P450s in tumors. The enrichment of IL‐6 in colonic inflammation can upregulate the expression of CYP2E1 and CYP1B1, thereby activating dietary carcinogens and DNA damage, thus promoting colorectal carcinogenesis.^48^ CYP1A1 is one of the newly discovered major metabolic enzymes of lenvatinib, and in some instances, its ability to form O‐desmethyl lenvatinib is stronger than other enzymes, including CYP3A4, the most important metabolic enzyme of lenvatinib.^49^ BGM is a competitive inhibitor of CYP1A1 and has been shown to inhibit different kinds of tumors.^50^, ^51^, ^52^ We performed a tumor‐bearing mouse model to prove that BGM can not only improve the efficacy of lenvatinib but also enhance the effect of combination therapy. After IHC was conducted on the liver and tumor tissue of mice, we found that the expression of CYP1A1 in tumor tissue increased significantly after treatment, while the change in the liver tissue was not obvious. This phenomenon, combined with the heterogeneity of CYP1A1 expression in tumors mentioned previously, indicates the active metabolic reprogramming in tumor tissue, particularly during combination therapy. The heterogeneity and plasticity of drug metabolism in hepatoma cells suggest that in some cases, tumor cells may metabolize more drugs than normal liver tissue especially when the tumor burden is high enough, leading to alterations in drug concentrations in the blood and local tumor areas. The impact of this phenomenon on antitumor therapies merits further investigation.

**FIGURE 8:**
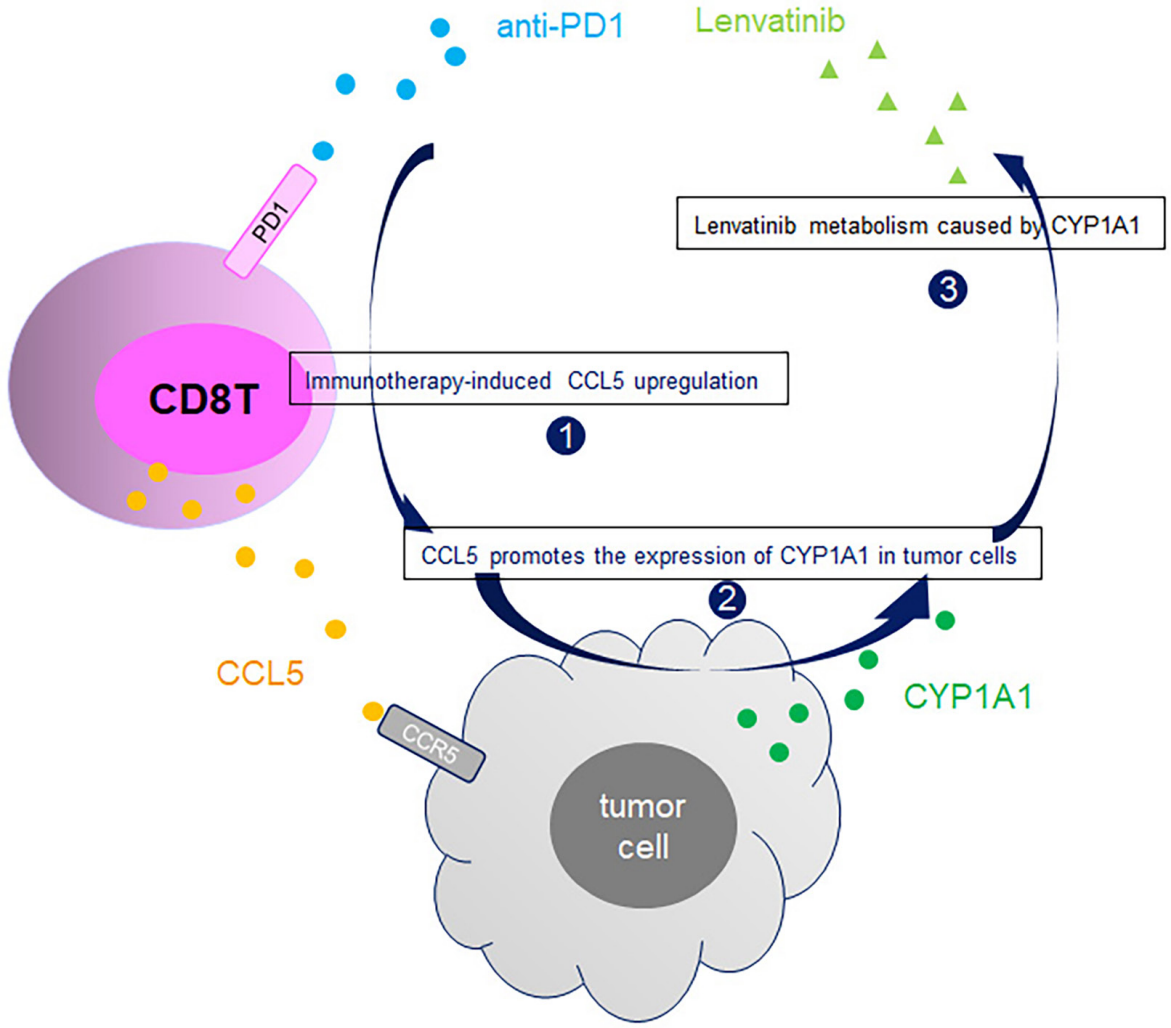
Sketch map: the CCL5/CCR5/CYP1A1 pathway is activated during combination therapy, which promotes resistance to lenvatinib in hepatocellular carcinoma cells.

In conclusion, our study suggests that the CCL5/CCR5/CYP1A1 pathway is activated during combination therapy to mediate metabolic reprogramming of liver cancer cells to resist lenvatinib (Figure 8). Interference with this pathway is expected to improve the efficacy of levatinib or combination therapy.

## AUTHOR CONTRIBUTIONS

**Yafei Wang:** Data curation; formal analysis; investigation; methodology; resources; software; validation; writing – original draft. **Biao Gao:** Formal analysis; methodology; software. **Tianyu Jiao:** Methodology; visualization. **Wenwen Zhang:** Investigation; methodology; resources. **Huizhong Shi:** Methodology. **Hao Jiang:** Methodology. **Xuerui Li:** Resources. **Junfeng Li:** Resources. **Xinlan Ge:** Methodology. **Ke Pan:** Methodology. **Chonghui Li:** Investigation; project administration; writing – review and editing. **Guankun Mao:** Conceptualization; methodology; project administration. **Shichun Lu:** Conceptualization; project administration; resources; supervision.

## FUNDING INFORMATION

This research did not receive any specific grant from funding agencies in the public, commercial, or not‐for‐profit sectors.

## CONFLICT OF INTEREST STATEMENT

The authors declare that the research was conducted in the absence of any commercial or financial relation.

## ETHICS STATEMENT

Approval of the research protocol by an Institutional Review Board: Yes.

Informed Consent: All patients provided their signatures on the written informed consent document.

Registry and the Registration No. of the study/trial: The study was approved by the Research Ethics Committee of Chinese PLA General Hospital (Beijing, China) (S2018‐111‐01) and implemented in accordance with the Helsinki Declaration of Principles.

Animal Studies: All animal studies adhered to the guidelines established by the institution's ethical committee.

## Supporting information


Appendix S1.

Appendix S2.

## Data Availability

The RNA‐seq data on CCL5‐stimulated Huh7 cells has been deposited in Gene Expression Omnibus (GEO) with accession number GSE246378 (Private until Oct 30, 2023). The scRNA‐seq data from the samples before and after combination therapy is available on reasonable request.
