# Supplementary material for: CCL5/CCR5/CYP1A1 pathway prompts liver cancer cells to survive in the combination of targeted and immunological therapies
Source: Cancer Sci. 2024 Aug 25;115(11):3552–69. doi: 10.1111/cas.16320 (PMC11531955; doi:10.1111/cas.16320)
Supplement: Supplementary file 1 — Appendix S1. [file CAS-115-3552-s002.docx]

**Supplementary Figures Here**


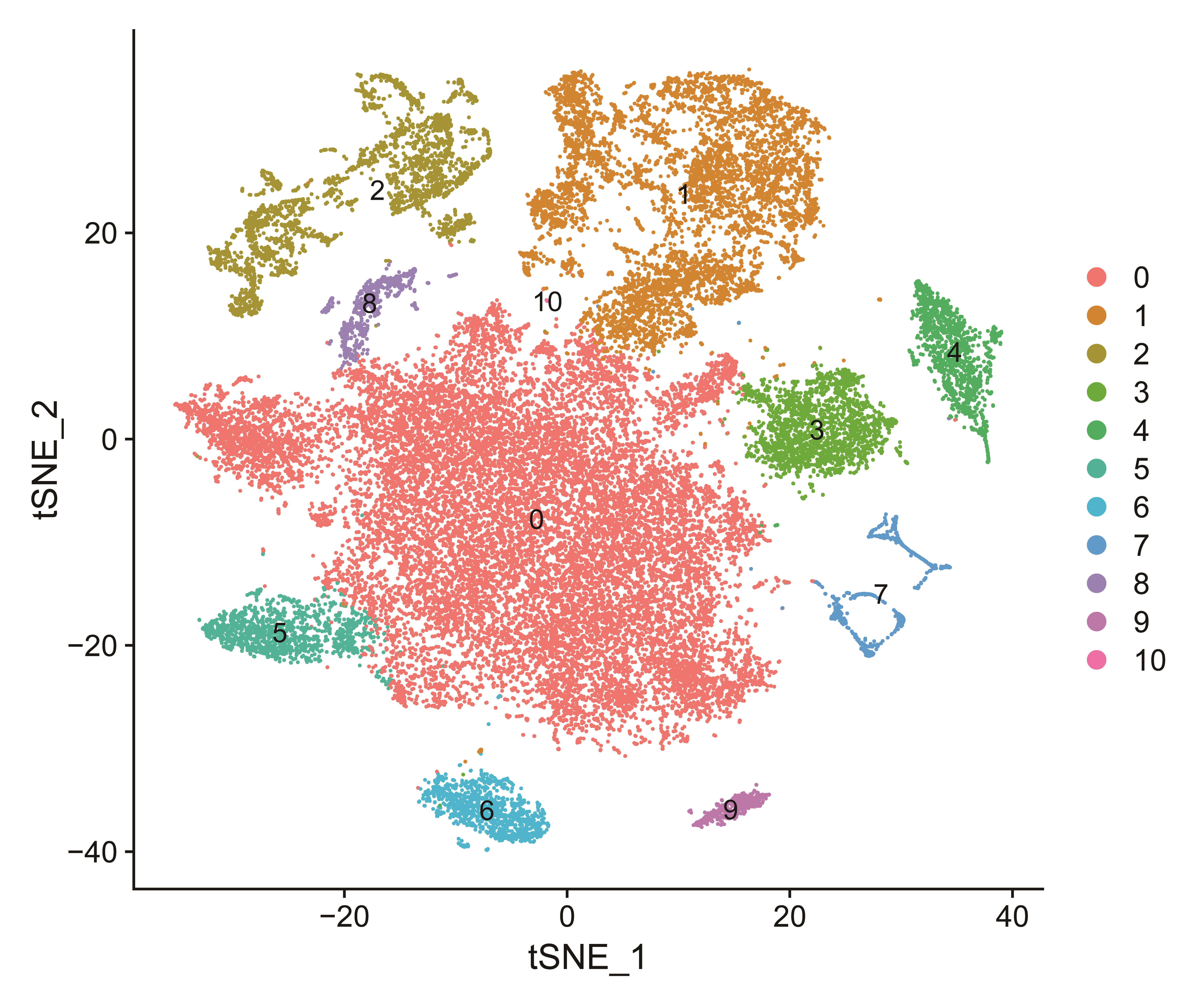


**Figure S1.** t-SNE plot showing eleven distinct cell clusters for 34512 high-quality single cells from samples before or after combination therapy.


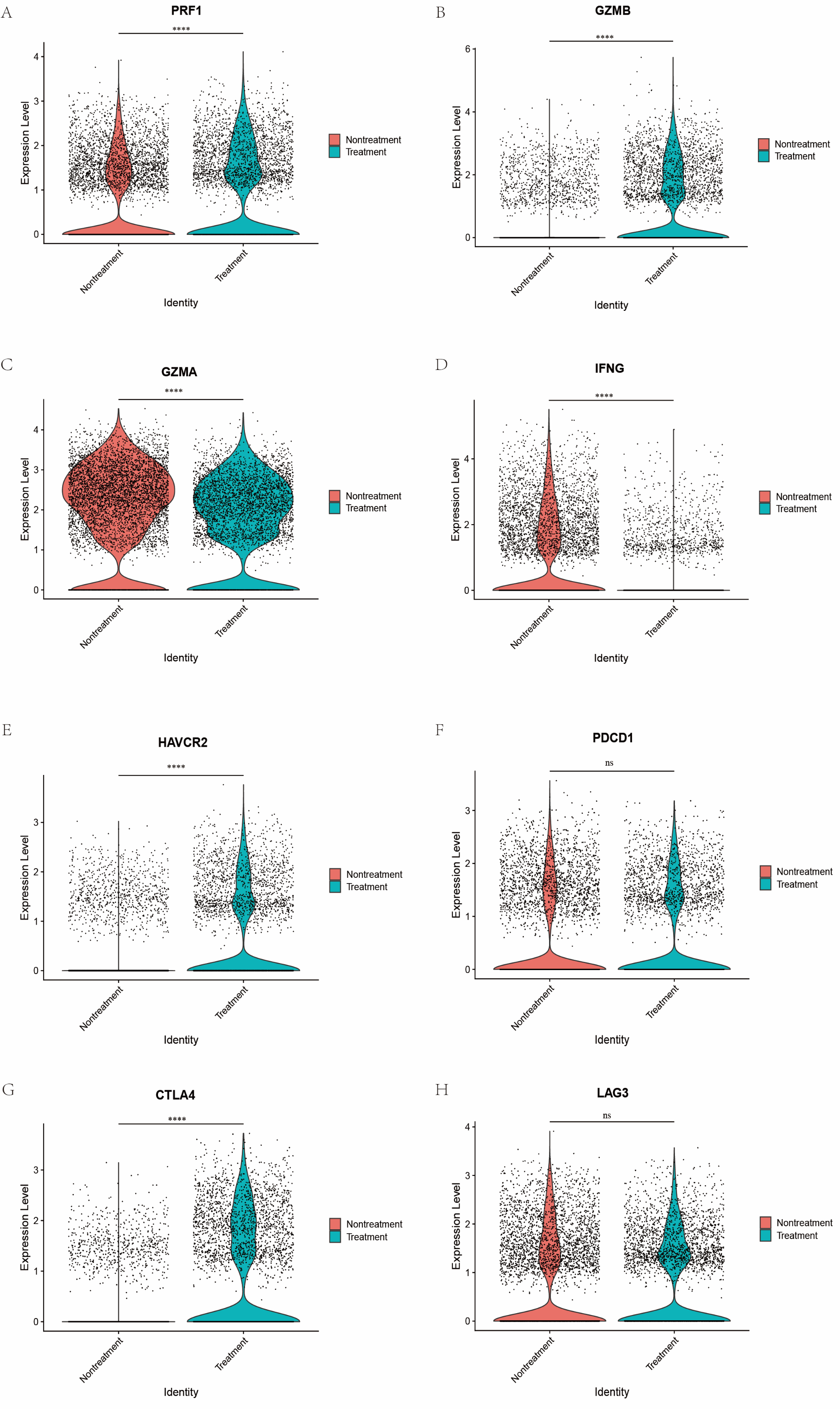


**Figure S2.** Expression of cytotoxic (A-D) and immunosuppressive (E-H) genes in exhausted CD8T cells before and after combined treatment.


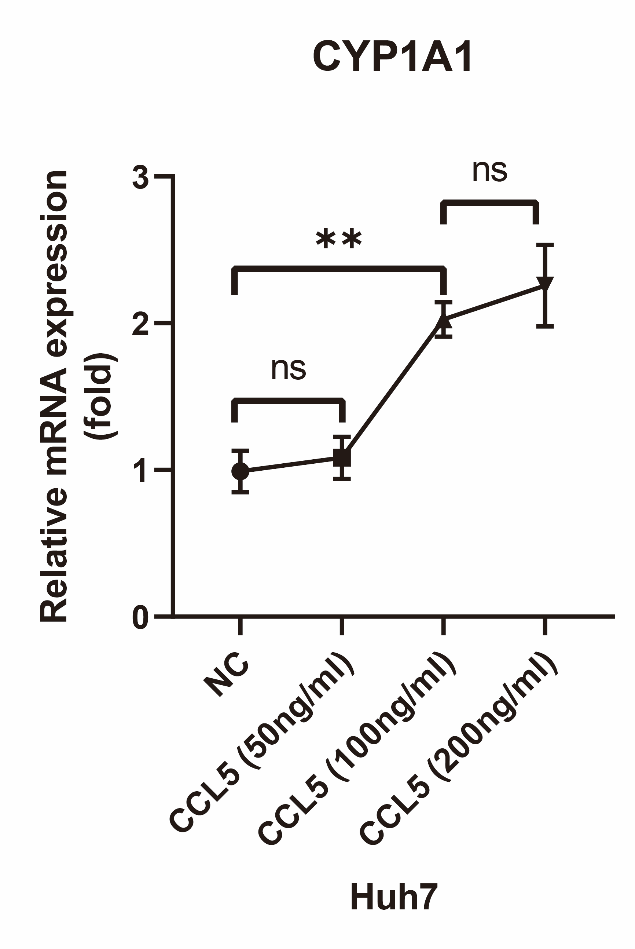


**Figure S3.** CYP1A1 expression in Huh7 cells after stimulation with different concentrations of CCL5 protein.


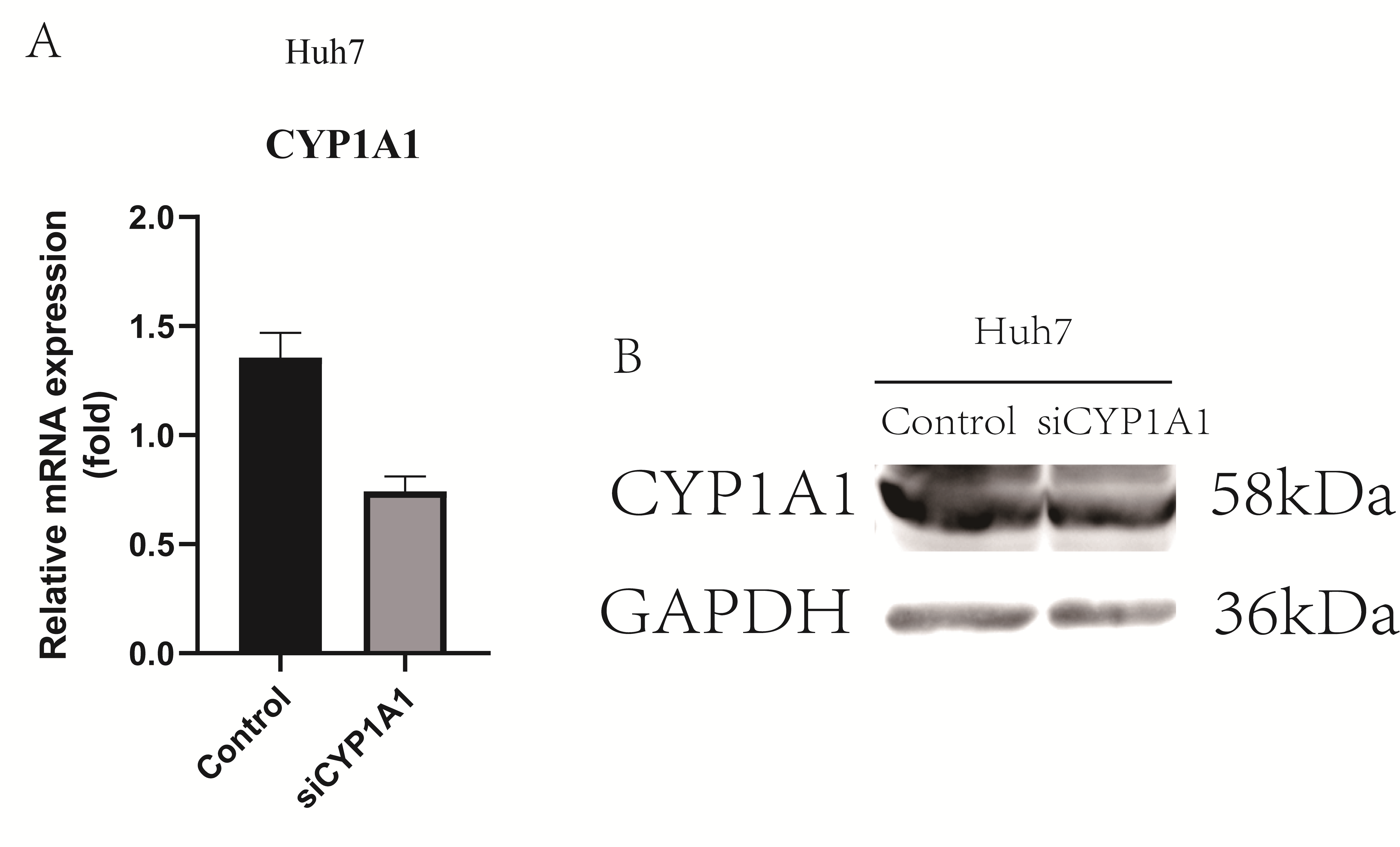


**Figure S4.** (A)qPCR (B)Western Blotting (WB) analysis showing the interference effect of siCYP1A1 on huh7 cell line


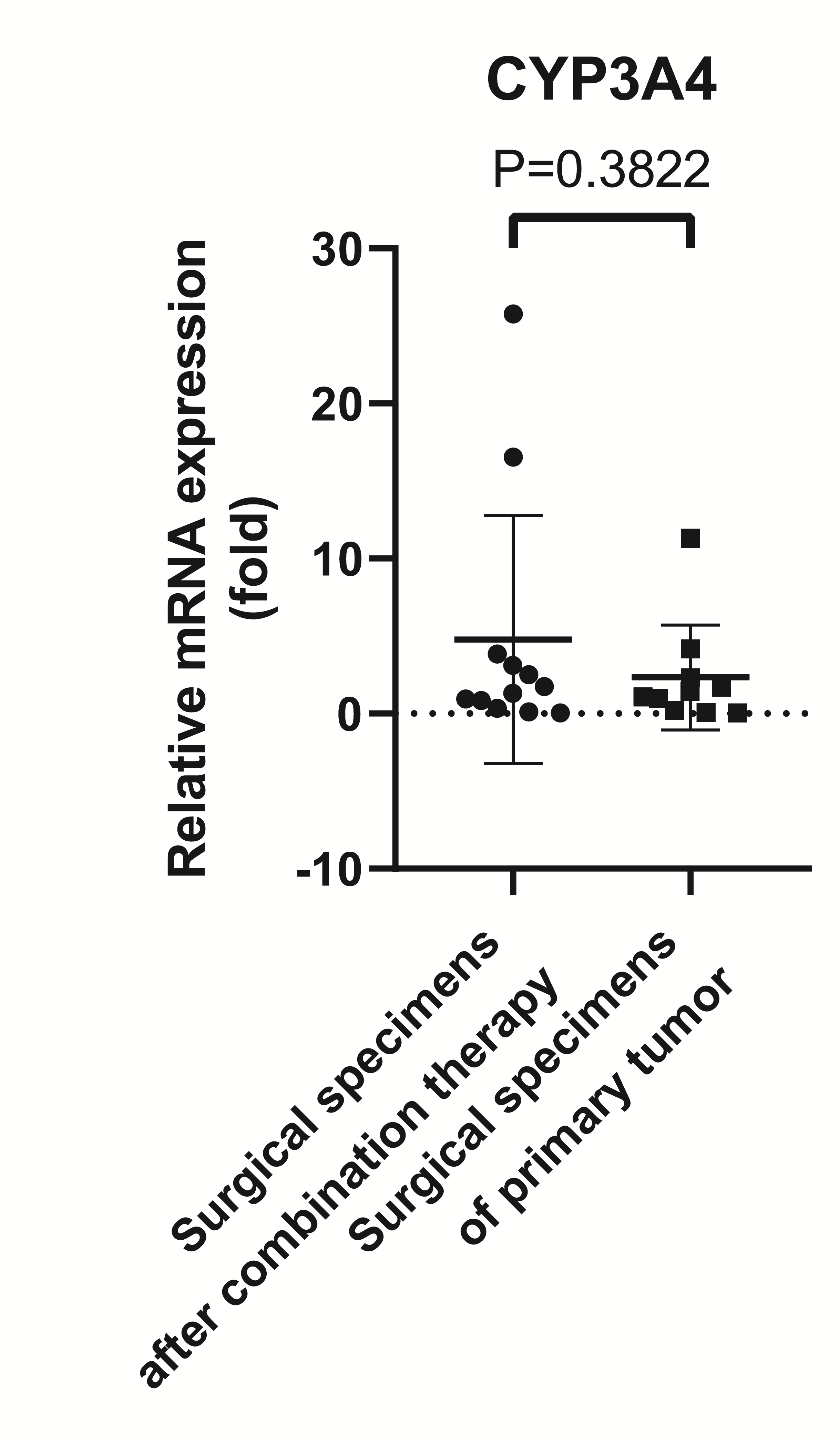


**Figure S5.** The mRNA level of CYP3A4 in residual tumor tissue before and after combination therapy.


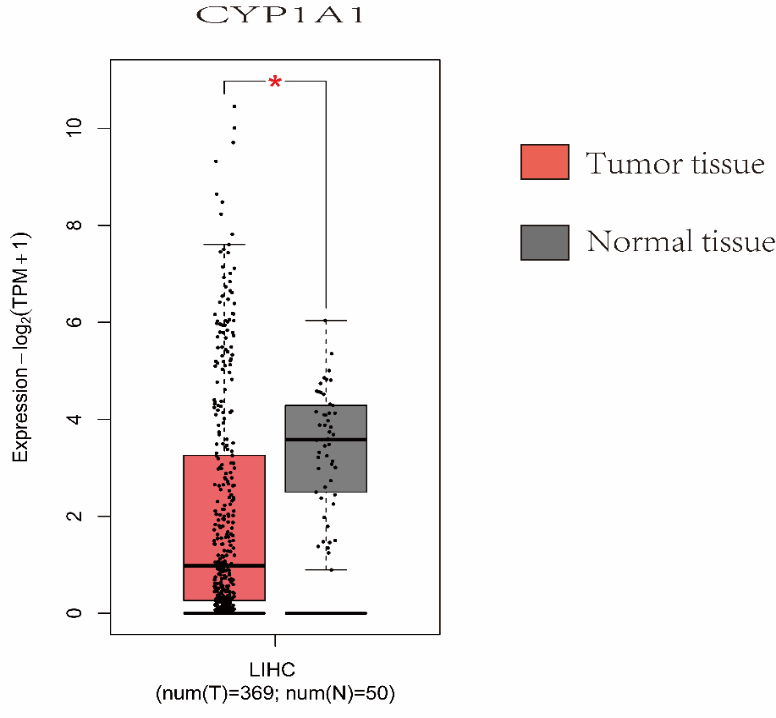


**Figure S6.** The mRNA levels of CYP1A1 in tumor tissue and normal tissue in HCC.


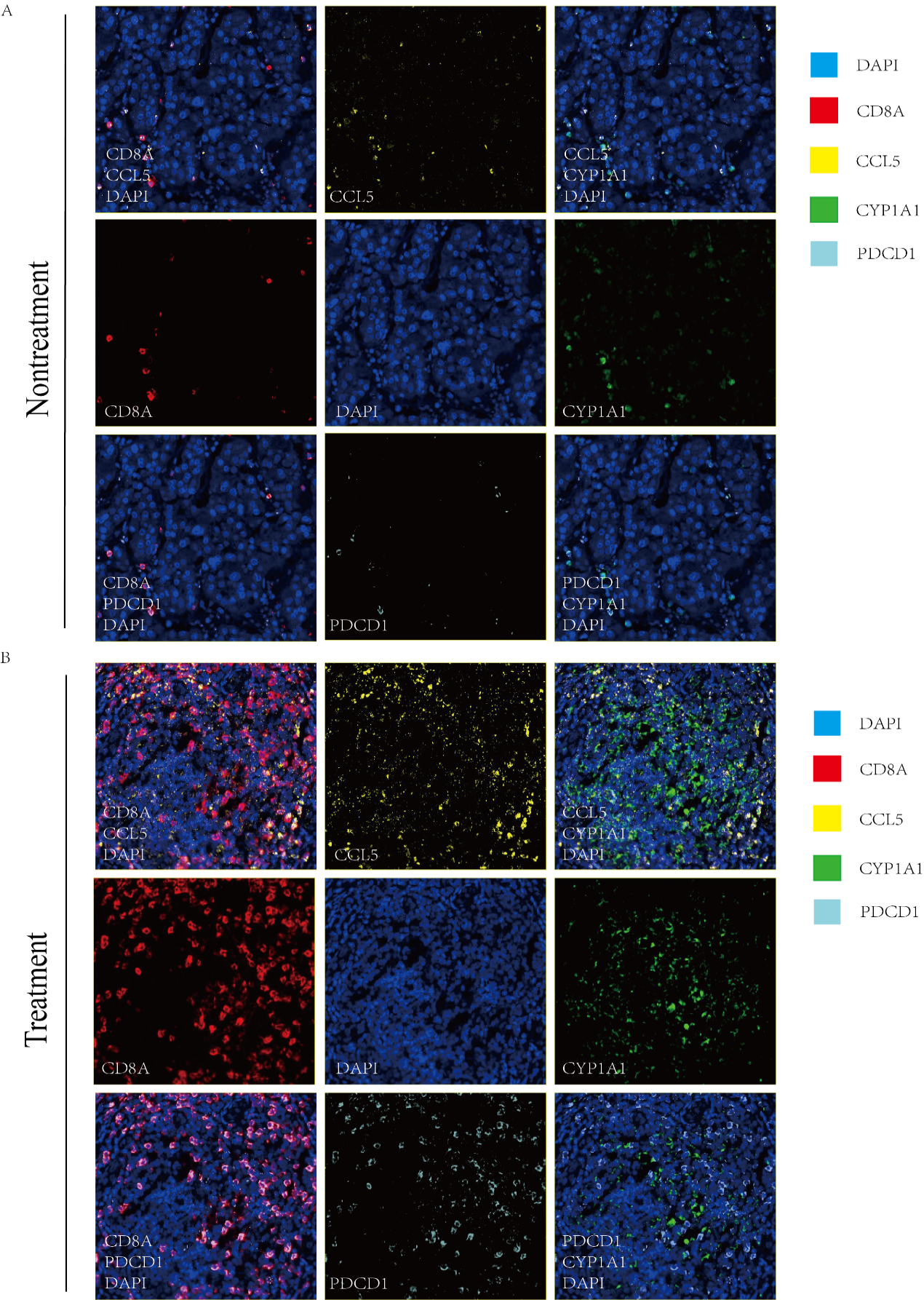


**Figure S7.** mIHC showing the expression of CCL5, CD8A, PDCD1, and CYP1A1 in surgical specimens from patients without (A) or with (B) combination therapy. Nontreatment: HCC specimens without combination therapy; Treatment: HCC specimens after combination therapy.


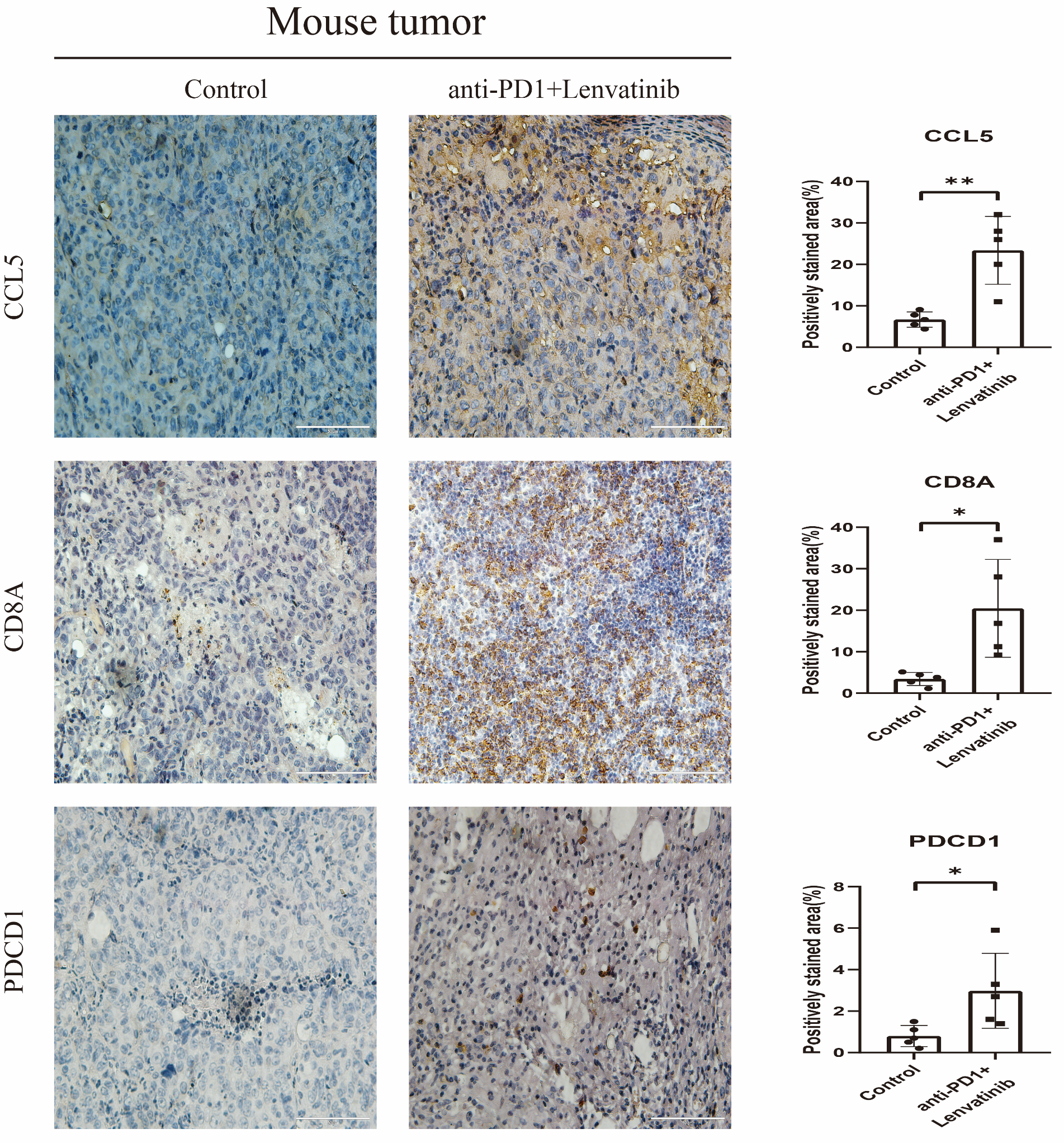


**Figure S8.** Expression of CCL5, CD8A, and PDCD1 of mouse tumor in the control group and combination therapy (anti-PD1 plus lenvatinib) group.

**Supplementary Tables Here**

| Table S1. The reagents and materials used in our study | | |  |  |  |
| --- | --- | --- | --- | --- | --- |
| [**Name**](javascript:;) | | | [**Brand**](javascript:;) | **Country** |  |
| Hepa 1-6 (Mouse liver Cancer cells) | | | Procell | China |  |
| Hep 3B2.1-7(Human liver Cancer cells) | | | Procell | China |  |
| HuH-7 (Human liver Cancer cells) | | | Procell | China |  |
| Hep G2(Human liver Cancer cells) | | | Procell | China |  |
| DMEM | | | Solarbio | China |  |
| FBS | | | ExcellBio | China |  |
| RANTES/CCL5, Human (HEK293) | | | MCE | USA |  |
| CCL5-mouse | | | MCE | USA |  |
| Maraviroc | | | CHEMEGEN | USA |  |
| Bergamottin | | | MCE | USA |  |
| Lenvatinib | | | MCE | USA |  |
| Lenvatinib Mesilate Capsules | | | Lenvima | Canada |  |
| Anti-CD8 antibody | | | ZSGB-BIO | China |  |
| PD-1 (EH33) Mouse mAb | | | CST | USA |  |
| Polyclonal Rabbit anti‑Human CCL5 / RANTES Antibody | | | Lsbio | USA |  |
| InVivoMAb anti-mouse PD-1 (CD279) | | | BioXCell | USA |  |
| *InVivo*MAb polyclonal Armenian hamster IgG | | | BioXCell | USA |  |
| Anti-RANTE antibody | | | Abcam | UK |  |
| Anti-CCR5 antibody | | | Abcam | UK |  |
| CYP1A1 Polyclonal Antibody | | | Thermofisher | USA |  |
| Anti-CYP1A1 antibody | | | Abcam | USA |  |
| Cell Counting Kit-8 | | | Solarbio | China |  |
| GAPDH mouse mAb | | | SUNGENE BIOTECH | China |  |
| β-Actin mouse mAb | | | SUNGENE BIOTECH | China |  |
| Horseradish Enzyme-labeled Goat Anti-Rabbit IgG (H+L) | | | ZSGB-BIO | China |  |
| Horseradish Enzyme-labeled Goat Anti-Mouse IgG (H+L) | | | ZSGB-BIO | China |  |
| Opal 6-Plex Manual Detection Kit | | | Akoya | USA |  |
| 2×RealStar Fast SYBR qPCR Mix (High ROX) | | | GenStar | China |  |
| StarScript II RT MasterMix | | | GenStar | China |  |
| RNAsimple Total RNA Kit | | | TIANGEN | China |  |
| RIPA Lysis Buffer | | | GenStar | China |  |
| Super ECL Plus | | | Huaxingbio | China |  |
| RNAfit | | | HANbio | China |  |
| SweTransDNA | | | Servicebio | China |  |
| anti-rabbit Alexa Fluor® 594 | | | Jackson | USA |  |
| anti-mouse Alexa Fluor® 488 | | | Jackson | USA |  |
| Table S2. The forward and revere primers used in qPCR analysis. | | | | | |
| Gene | Forward primer | Reverse primer | | | |
| CCL5 | TCCTGCATCTGCCTCCCCATATT | CCGAACCCATTTCTTCTCTGGGTT | | | |
| CCR5 | ACATGCTGGTCATCCTCATCCT | CACCACCCAAGTGATCACACTTGT | | | |
| CYP1A1 | TTCTGGCCTCTGTCATCTTCTGTC | GCTGAAGGACATGCTCTGACCATT | | | |
| CYP3A4 | AGACTTGGCCATGGAAACCTG | GCAATGATAGGGACCATCTCCTTG | | | |
| ACTB | GCATGGGTCAGAAGGATTCCTATGT | CGGTGAGGATCTTCATGAGGTAGT | | | |
| GAPDH | GAGTCAACGGATTTGGTCGTATTGG | ACTTGATTTTGGAGGGATCTCGCT | | | |

Table S3.Information on patients with pathologically confirmed residual tumors and degree of tumor differentiation

| **Patient_ID** | **Gender** | **Age** | **BCLC Stage** | **AFP(ng/ml)** | **History of viral hepatitis** | **anti-PD1 antibody** | [**Targeted drug**](https://www.webofscience.com/wos/alldb/full-record/WOS:001060664700001) |
| --- | --- | --- | --- | --- | --- | --- | --- |
| Patient_1 | male | 33 | C | 1534 | HBV | Pembrolizumab | Lenvatinib |
| Patient_2 | female | 67 | C | 2.89 | HCV | Pembrolizumab | Lenvatinib |
| Patient_3 | female | 28 | C | 1.7 | None | Sintilimab | Lenvatinib |
| Patient_4 | male | 56 | C | >3000 | HCV | Tislelizumab | Lenvatinib |
| Patient_5 | male | 55 | C | 3.14 | HBV | Sintilimab | Lenvatinib |
| Patient_6 | female | 73 | C | 2.29 | None | Sintilimab | Lenvatinib |
| Patient_7 | male | 52 | C | 4.63 | HBV | Sintilimab | Lenvatinib |
| Patient_8 | male | 53 | C | 13.97 | HBV | Treprinumab | apatinib |
| Patient_9 | male | 54 | C | 9.98 | HBV | Pembrolizumab | Lenvatinib |
| Patient_10 | male | 33 | C | 20.91 | HBV | Sintilimab | Lenvatinib |
| Patient_11 | male | 32 | C | 789 | HBV | Sintilimab | Lenvatinib |
| Patient_12 | female | 54 | C | 3.53 | None | Sintilimab | Lenvatinib |
| Patient_13 | male | 57 | B | 772 | None | Treprinumab | Lenvatinib |
| Patient_14 | male | 70 | B | 11.96 | None | Sintilimab | Lenvatinib |
| Patient_15 | male | 55 | C | 2.92 | None | Sintilimab | Lenvatinib |
| Patient_16 | female | 56 | C | 7.02 | HBV | Sintilimab | Lenvatinib |
| Patient_17 | male | 47 | C | 12.67 | HBV | Sintilimab | Lenvatinib |
| Patient_18 | male | 49 | C | 20225 | HBV | Sintilimab | Lenvatinib |
| Patient_19 | male | 41 | C | 25.1 | HBV | Sintilimab | Lenvatinib |
| Patient_20 | male | 55 | C | 54.73 | HCV | Sintilimab | Lenvatinib |
| Patient_21 | male | 66 | C | 22.9 | HCV | Sintilimab | Lenvatinib |
| Patient_22 | male | 65 | C | 20.6 | HBV | Sintilimab | Lenvatinib |
| Patient_23 | male | 67 | C | 121 | HBV | Sintilimab | Lenvatinib |
| Patient_24 | male | 58 | C | 34.1 | HBV | Sintilimab | Lenvatinib |
| Patient_25 | male | 43 | C | >60500 | HBV | Sintilimab | Lenvatinib |
| Patient_26 | male | 62 | A | 0.68 | None | Sintilimab | Lenvatinib |
| Patient_27 | male | 52 | C | 165.4 | HBV | Treprinumab | Lenvatinib |
| Patient_28 | male | 73 | B | 0 | HBV | Sintilimab | Lenvatinib |
| Patient_29 | female | 54 | C | 3.53 | None | Sintilimab | Lenvatinib |
| Patient_30 | male | 57 | B | 777.2 | None | Treprinumab | Lenvatinib |
| Patient_31 | male | 57 | C | 85.6 | HBV | Sintilimab | Lenvatinib |
| Patient_32 | female | 73 | C | 10.92 | None | Sintilimab | Lenvatinib |
| Patient_33 | male | 54 | C | 34.42 | HBV | Sintilimab | Lenvatinib |
| Patient_34 | female | 44 | C | 6917 | HBV | Sintilimab | Lenvatinib |
| Patient_35 | male | 51 | C | 3 | HBV | Sintilimab | Lenvatinib |
| Patient_36 | male | 54 | C | 492.4 | HBV | Sintilimab | Lenvatinib |
| Patient_37 | female | 44 | C | 4457 | HBV | Sintilimab | Lenvatinib |
| Patient_38 | male | 55 | C | 8.38 | HBV | Pembrolizumab | Lenvatinib |
| Patient_39 | male | 54 | C | 2859 | HBV | Sintilimab | Lenvatinib |
| Patient_40 | male | 61 | C | >60500 | HBV | Sintilimab | Lenvatinib |
| Patient_41 | male | 50 | C | 1800 | HBV | Treprinumab | Lenvatinib |
| Patient_42 | male | 24 | C | 451.8 | HBV | Treprinumab | Lenvatinib |
| Patient_43 | male | 61 | C | 7058 | HCV | Sintilimab | Lenvatinib |
| Patient_44 | male | 53 | C | 24246 | HCV | Tislelizumab | Lenvatinib |
| Patient_45 | male | 42 | C | >60500 | HBV | Sintilimab | Lenvatinib |
| Patient_46 | female | 46 | C | 9995 | HBV | Sintilimab | Lenvatinib |
| Patient_47 | male | 43 | C | 436 | HBV | Sintilimab | Lenvatinib |
| Patient_48 | male | 38 | C | 289 | HBV | Sintilimab | Lenvatinib |
| Patient_49 | female | 63 | C | 15265 | HBV | Sintilimab | Lenvatinib |
| Patient_50 | male | 50 | C | 17008 | HBV | Sintilimab | Lenvatinib |
| Patient_51 | male | 31 | C | 2660 | HBV | Tislelizumab | Lenvatinib |
| Patient_52 | male | 56 | C | 3.19 | HBV | Tislelizumab | Lenvatinib |
| Patient_53 | male | 58 | A | >60500 | HBV | Sintilimab | Lenvatinib |
| Patient_54 | male | 46 | C | 37950 | HBV | Sintilimab | Lenvatinib |
| Patient_55 | male | 47 | C | 973.6 | HBV | Sintilimab | Lenvatinib |
| Patient_56 | male | 54 | C | 121 | HBV | Sintilimab | Lenvatinib |
| Patient_57 | male | 55 | C | 1869 | HBV | Sintilimab | Lenvatinib |
| Patient_58 | male | 64 | B | >60500 | HBV | Sintilimab | Lenvatinib |
| Patient_59 | male | 58 | C | 0 | HBV | Sintilimab | Lenvatinib |
| Patient_60 | male | 53 | B | 46.01 | HBV | Sintilimab | Lenvatinib |

| **Patient_ID** | **Cycles of combination therapy** | **The proportion of residual tumors(%)** | **Pathological differentiation grade** |
| --- | --- | --- | --- |
| Patient_1 | 4 | 80 | G1/2 |
| Patient_2 | 4 | 100 | G1/2 |
| Patient_3 | 3 | 80 | G1/2 |
| Patient_4 | 3 | 85 | G1/2 |
| Patient_5 | 4 | 50 | G1/2 |
| Patient_6 | 4 | 50 | G1/2 |
| Patient_7 | 4 | 50 | G1/2 |
| Patient_8 | 3 | 3 | G1/2 |
| Patient_9 | 7 | 95 | G1/2 |
| Patient_10 | 4 | 90 | G1/2 |
| Patient_11 | 4 | 90 | G1/2 |
| Patient_12 | 4 | 50 | G1/2 |
| Patient_13 | 3 | 85 | G1/2 |
| Patient_14 | 3 | 89 | G1/2 |
| Patient_15 | 3 | 80 | G1/2 |
| Patient_16 | 6 | 20 | G1/2 |
| Patient_17 | 3 | 50 | G1/2 |
| Patient_18 | 4 | 70 | G1/2 |
| Patient_19 | 6 | 8 | G1/2 |
| Patient_20 | 7 | 100 | G1/2 |
| Patient_21 | 7 | 35 | G1/2 |
| Patient_22 | 3 | 100 | G1/2 |
| Patient_23 | 6 | 80 | G1/2 |
| Patient_24 | 5 | 40 | G1/2 |
| Patient_25 | 3 | 10 | G1/2 |
| Patient_26 | 5 | 70 | G1/2 |
| Patient_27 | 5 | 30 | G1/2 |
| Patient_28 | 4 | 42 | G1/2 |
| Patient_29 | 4 | 50 | G1/2 |
| Patient_30 | 3 | 85 | G1/2 |
| Patient_31 | 5 | 45 | G1/2 |
| Patient_32 | 4 | 50 | G1/2 |
| Patient_33 | 4 | 70 | G1/2 |
| Patient_34 | 6 | 5 | G3/4 |
| Patient_35 | 3 | 5 | G3/4 |
| Patient_36 | 3 | 2 | G3/4 |
| Patient_37 | 5 | 5 | G3/4 |
| Patient_38 | 3 | 2.5 | G3/4 |
| Patient_39 | 6 | 10 | G3/4 |
| Patient_40 | 5 | 15 | G3/4 |
| Patient_41 | 10 | 50 | G3/4 |
| Patient_42 | 14 | 10 | G3/4 |
| Patient_43 | 4 | 20 | G3/4 |
| Patient_44 | 5 | 20 | G3/4 |
| Patient_45 | 3 | 70 | G3/4 |
| Patient_46 | 4 | 90 | G3/4 |
| Patient_47 | 3 | 30 | G3/4 |
| Patient_48 | 9 | 85 | G3/4 |
| Patient_49 | 3 | 60 | G3/4 |
| Patient_50 | 3 | 100 | G3/4 |
| Patient_51 | 6 | 30 | G3/4 |
| Patient_52 | 3 | 15 | G3/4 |
| Patient_53 | 5 | 10 | G3/4 |
| Patient_54 | 7 | 80 | G3/4 |
| Patient_55 | 5 | 10 | G3/4 |
| Patient_56 | 7 | 9 | G3/4 |
| Patient_57 | 5 | 1 | G3/4 |
| Patient_58 | 5 | 20 | G3/4 |
| Patient_59 | 6 | 10 | G3/4 |
| Patient_60 | 4 | 100 | G3/4 |
