# Supplementary material for: CCL5/CCR5/CYP1A1 pathway prompts liver cancer cells to survive in the combination of targeted and immunological therapies
Source: Cancer Sci. 2024 Aug 25;115(11):3552–69. doi: 10.1111/cas.16320 (PMC11531955; doi:10.1111/cas.16320)
Supplement: Supplementary file 2 — Appendix S2. [file CAS-115-3552-s001.docx]

**Supplenmentary Methodologies Here**

**1.Supplementary methodology for scRNA-seq**

**Tissue dissociation and cell purification** The freshly collected samples were promptly dispatched to the research facility without delay. Subsequently, the MACS Tumour Dissociation Kit was employed in strict adherence to the guidelines provided by the manufacturer. Fresh tumor tissue was carefully sliced into about 1 mm^3^ fragments, which were then immersed in RPMI-1640 culture supplemented with 10% fetal bovine serum (FBS). The enzymatic dissociation process was carried out for 30 minutes at a temperature of 37°C, employing a rotator for agitation. After the filtration procedure utilizing a 70μm cell strainer, the cells in suspension were subjected to centrifugation at 400g for five minutes. After performing a double phosphate-buffered saline (PBS) wash and removing the supernatant, the cellular particles were re-suspended in a sorting buffer. Following the isolation of single-cell suspensions from different samples, an automated counter was employed to determine the cell count per milliliter of suspension. The concentration and viability of cells were subsequently assessed by a Rigel S3 fluorescent cell analyzer (Countstar, USA). To create single-cell gel beads in reagents, cells were put into a Chromium Single Cell Controller (10x Genomics, USA) under the manufacturer's instructions. The scRNA-seq libraries were constructed using the Single Cell 3′ Library and Gel Bead Kit v3.1. A NovaSeq6000 sequencer (Illumina, USA) was used to sequence the produced libraries.

**scRNA-seq data processing** The scRNA-seq data were analyzed and quantified against the human reference genome GRCh38 using the CellRanger v.3.1 toolset. The analysis of gene expression matrices for each sample was conducted using the Seurat package (version 3.2.2) in R software (version 4.2). To eliminate cells of inferior quality and doublets (i.e., two cells enclosed within a single droplet) for each sample, cells that had either fewer than 200 unique molecular identifiers (UMIs), or over 8000 or below 200 expressed genes, were removed. To filter out dead or dying cells, the cells that had over 10% UMIs derived from the mitochondrial genome were further removed. This resulted in a total of 34,512 high-quality viable cells in all samples. Gene expression was scaled after being normalized with the "LogNormalize" method. Using Seurat's "FindVariableGene" function, 2000 highly variable genes (HVGs) were identified following data normalization. The distribution of P-values was visualized using the "JackStraw" and "ScoreJackStraw" functions after PCA was used to identify significant principal components (PCs). Twenty PCs were chosen for t-SNE analysis in the end. The K-nearest neighbours (KNN) analysis and the "FindClusters" algorithm were employed to partition the cells into 11 distinct clusters, with a resolution of 0.1.

**Cell clustering and annotation** The cell clusters were annotated using known genes: T-cells (CD3D, CD3E, CD8A, CCR5), B-cells (CD19, CD79A, and CD79B), myeloid cells (APOC1, APOE, CCR2), CAFs (SPARC, BGN, COLEC11) and mast cells (HDC, KIT, TPSB2), epithelial cells (ALB, AFP, APOA2, ARG1).

**Differential gene expression analysis** The “FindAllMarkers” function was used to calculate the marker genes for each cell cluster with the following parameters: positive markers only, proportion of expressed cells within the cluster ≥ 0.25, difference in the proportion of expressed cells within and outside the cluster ≥ 0.25, logfc.threshold > 1.

**Functional enrichment analysis** The Limma package of R software was utilized to identify genes that were differentially expressed between cells before and after combination therapy. The ClusterProfile package of the R software was employed to investigate the set of genes that exhibited substantial enrichment in each cell cluster. These genes were further filtered based on the criterion of adj. p value < 0.05.

**Pseudotime analysis** R software's Monocle2 package was used to deduce the CD8+ T cells' pseudotime trajectory. In order to reduce dimensionality, we employed the “DDRTree” methodology to arrange cells according to the 1000 genes exhibiting the most significant alterations.

**2. Supplementary methodology for cell culture, stimulation and cell transfection**

The human hepatoma cell lines HepG2, Hep3B, Huh7, and the mouse hepatoma cell line Hepa 1-6 were cultured in Dulbecco's Modified Eagle’s Medium (DMEM) supplemented with 10% fetal bovine serum (FBS), without the presence of antibiotics. The cell lines were maintained in a humidified incubator at a temperature of 37 ℃ with 5% CO2. The concentrations of CCL5, maraviroc (MVC), and lenvatinib used for cell stimulation were 100ng/ml, 200ng/ml, and 100ng/ml, respectively. Corresponding control groups were stimulated with the same volume of solvent. The si-CYP1A1 (5’-CAGATGAGAAGATCATTAACA-3’) used for cell transfection was synthesized by Tsingke Biotech (Beijing, China). Transfection was then conducted for 6 hours via RNAfit in accordance with the provided instructions. The concentration of si-CYP1A1 utilised for transfection in this study was 50nM. After replacing the medium, the cell culture was continued for 24 hours, and subsequent experiments were then started.

**3. Supplementary methodology for RNA-seq Analysis**

24 hours after CCL5 stimulation, huh7 cells were washed with PBS and lysed with TRIzol for RNA extraction. The RNA samples were processed for RNA-seq library construction using the VAHTS Universal V6 RNA-seq Library Prep Kit for MGI® (Vazyme, NRM604-01) and subsequently sequenced with DNBSEQ T7 platform (MGI).

**4. Supplementary methodology for qPCR**

After different stimulations for 24 hours, the RNA of the cell lines was extracted using the RNAsimple Total RNA Kit. Subsequently, cDNA synthesis was performed through reverse transcription utilizing the StarScript II RT MasterMix. qPCR analysis was carried out employing 2×RealStar Fast SYBR qPCR Mix (High ROX) and detection was accomplished using a StepOnePlus Real-Time PCR System (ABI, USA). Quantitative analysis of mRNA expression was conducted using the 2^-△△Ct^ method with normalization against housekeeping genes (ACTB).

24 frozen tumor samples, including 12 samples after combination therapy and 12 specimens of direct surgeries, were swiftly homogenized upon the addition of lysis buffer, facilitating the extraction of RNA. Subsequently, the homogenates were left at room temperature for 5 minutes before being centrifuged to obtain supernatants. The subsequent steps adhered to the procedures mentioned above.

The forward and reveres primers used in this study are detailed in Table2 .

**5. Supplementary methodology for Western Blotting (WB) analysis**

48 hours after different stimulation, the cell lines were washed with PBS and lysed using RIPA lysis buffer for protein extraction. 25 frozen tumor samples, including 13 samples after combination therapy and 12 specimens of direct surgeries, were homogenized and lysed with RIPA lysis buffer. The lysis mixtures were then placed on ice for 30 minutes. Subsequently, the mixtures were centrifuged at high speed (12000 rpm/min) and low temperature (4°C) for 15 minutes to collect the supernatant. Prior to use, the RIPA lysis buffer was pre-mixed with protease inhibitors and phosphatase inhibitors. Throughout the entire protein extraction process, efforts were made to maintain a low temperature as much as possible. The BCA kit was used to determine protein concentration. Protein samples were diluted in RIPA buffer to a uniform concentration. An appropriate volume of 5×SDS-PAGE Sample Loading Buffer was added and thoroughly mixed with the protein samples. Then, the protein samples were denatured by boiling in water for 10 minutes. Extracted protein samples (15μg) underwent gel electrophoresis on SDS/PAGE followed by transfer onto PVDF membranes. After blocking with a solution of 5% skimmed milk powder for 1 hour at room temperature, PVDF membranes were incubated overnight at 4°C with primary antibodies (target proteins' antibodies at a dilution of 1:1000; internal reference proteins' antibodies at a dilution of 1:5000). Following this step, secondary antibodies (diluted at 1:10000) were applied to the membranes for an additional hour at room temperature. Protein signals were visualized in a dark room of LAS-3000mini (Fujifilm, Japan) after Super ECL Plus Detection Reagent was added to the membranes. Finally, Image J software was used to perform relative quantification analysis based on grayscale values obtained from protein bands.

**6. Supplementary methodology for immunohistochemistry (IHC)**

12 pathologically confirmed moderately differentiated tumor specimens, including 6 after combination therapy and 6 of direct operations, were selected for immunohistochemistry (IHC). The formalin-impregnated tissue was embedded in paraffin and subsequently sectioned into 5-μm-thick slices. Following dewaxing, antigen retrieval was performed using a 0.01M citrate buffer in a microwave oven. The slices were then immersed in a 3% hydrogen peroxide solution at room temperature for 10 minutes to inhibit endogenous peroxidase activity. To block non-specific antigen epitopes, goat serum was applied to the slices and incubated at room temperature for 30 minutes. After overnight incubation with the primary antibody at 4℃, the slices were treated with a secondary antibody derived from goats and incubated at room temperature for 1 hour. DAB solution was prepared and added to the slices, holding at room temperature for 10 minutes. Subsequently, the slices were counterstained with hematoxylin, dehydrated, cleared, and sealed with neutral resins. Five randomly selected fields of view were observed and photographed under a light microscope to assess the positivity rate by calculating the percentage of positively stained areas.

**7. Supplementary methodology for multiplex immunohistochemistry/immunofluorescence (mIHC/IF)**

24 formalin-fixed paraffin-embedded (FFPE) tissue, including 8 tumor samples from direct surgery and 16 tumor specimens with different proportions of tumor remaining after combination therapy, were chosen for multiplex immunohistochemistry/immunofluorescence (mIHC/IF). Following the provided instructions, these tissue were subjected to immunostaining using anti-human antibodies targeting CD8, CCL5, and CYP1A1 with the Opal 6-Plex Manual Detection Kit along with DAPI counterstaining. Images were acquired using Vectra 3.0 Pathology Imaging System Microscope (Perkin-Elmer, USA) and analyzed using QuPath (v0.4.3).

**8. Supplementary methodology for CCK-8 Kit Assay**

The cell viability was assessed using a cell counting kit-8 (CCK-8) assay. Huh7 cells were cultured overnight in 96-well plates. After the cells adhered to the dish, various stimuli were applied. 48 hours later, CCK-8 reagent was added as per the instructions and incubated with huh7 cells at 37°C for 1 hour. The absorbance (OD) at 450 nm was measured using Multiskan Mk3 (Thermo, USA).

**9. Supplementary methodology for clone formation assay**

The cell proliferation ability was assessed through a clone formation experiment. A total of 500 Huh7 cells were incubated in each well of six-well plates and cultured for approximately one week until stable clones formed (consisting of 10 to 20 cells per clone). Following the addition of lenvatinib and/or siCYP1A1 to the plates, the cells were further incubated for an additional week. Subsequently, the cells were fixed with 4% paraformaldehyde for 15 minutes and stained with a 1% crystal violet solution for 20 minutes. After washing the cells with PBS, photographs were taken to visualize the formation of clones.

**10. Supplementary methodology for wound healing assay**

The Huh7 cells were seeded evenly and cultured in six-well plates with the complete medium in a humidified incubator maintained at 37 °C with 5% CO_2_ until cell confluence reached above 90% on the plate bottom. Cell monolayers were scratched using a 200 μL sterile pipette tip, and then rinsed three times with PBS to remove cell debris. Following the application of various stimuli, the cells were further cultured in DMEM supplemented with 1% FBS for 48 hours. Cell migration in the wound area was observed and digitally photographed at either 24 or 48 hours post-scratching. Wound healing was measured on the images and the migration ratio was calculated by the area of the original wound minus the area of the wound during healing divided by the area of the original wound.

**11. Supplementary methodology for dual immunofluorescence**

Patient-derived frozen tissue was sliced at a thickness of 8μm under a low temperature of -20 °C after embedding in an optimal cutting temperature compound (OCT). Following fixation with 4% paraformaldehyde, the slices were permeabilized by incubating with 0.5% Triton X-100 for 30 minutes. To block non-specific antigen epitopes, the slices were treated with 1% BSA and incubated at room temperature for 30 minutes. Subsequently, a combination of primary antibodies was applied to the slices and left overnight at 4℃ for incubation. After that, secondary antibodies (anti-rabbit Alexa Fluor® 594 and anti-mouse Alexa Fluor® 488) were added and incubated for an hour at room temperature. Finally, the slices were sealed using Mounting Media (with DAPI) and observed under a fluorescence microscope (Olympus, Japan).

**12. Supplementary methodology for mice model**

A total of 2×10^6^ Hepa1-6 cells in 100μl PBS were subcutaneously injected into 8-week-old male C57BL/6J mice weighing about 20 grams. Tumor volume (length×width^2^×½) was assessed every three days. Once tumors reached 200mm^3^, mice were randomly divided into groups to receive lenvatinib, bergamottin (BGM), anti-PD1 antibody, anti-PD1 + BGM, lenvatinib + BGM, lenvatinib + anti-PD1 antibody, lenvatinib + anti-PD1 antibody + BGM, or corresponding solvent. Lenvatinib was dissolved in 3% hydrochloric acid while BGM was firstly dissolved in 10% DMSO and then diluted with 90% corn oil. Anti-PD1 and IgG antibodies were diluted with PBS. The drugs were administered as follows: oral gavage of 5mg/kg lenvatinib daily; intraperitoneal injection of 5mg/kg anti-PD1 antibody or IgG every three days; intraperitoneal injection of 50mg/kg BGM daily. The experiment was planned to be terminated on day 15 after intervention for specimen collection. All animal studies adhered to the guidelines established by the institution's ethical committee.
